# Genome-Wide Comparisons Reveal Extensive Divergence Within the Lichen Photobiont Genus, *Trebouxia*

**DOI:** 10.1093/gbe/evae219

**Published:** 2024-10-30

**Authors:** Rosa Celia Poquita-Du, Jürgen Otte, Anjuli Calchera, Imke Schmitt

**Affiliations:** Insititute of Ecology, Evolution and Diversity, Goethe University Frankfurt, Frankfurt, Germany; Senckenberg Biodiversity and Climate Research Centre (SBiK-F), Frankurt, Germany; LOEWE Centre for Translational Biodiversity Genomics, Frankfurt, Germany; Senckenberg Biodiversity and Climate Research Centre (SBiK-F), Frankurt, Germany; Senckenberg Biodiversity and Climate Research Centre (SBiK-F), Frankurt, Germany; LOEWE Centre for Translational Biodiversity Genomics, Frankfurt, Germany; Insititute of Ecology, Evolution and Diversity, Goethe University Frankfurt, Frankfurt, Germany; Senckenberg Biodiversity and Climate Research Centre (SBiK-F), Frankurt, Germany; LOEWE Centre for Translational Biodiversity Genomics, Frankfurt, Germany

**Keywords:** green algal isolate, PacBio genome sequencing, comparative genomics, symbiosis, algal symbiont

## Abstract

The green algal genus *Trebouxia* is the most frequently encountered photobiont of the lichen symbiosis. The single-celled symbionts have a worldwide distribution, including all continents and climate zones. The vast, largely undescribed, diversity of *Trebouxia* lineages is currently grouped into four phylogenetic clades (A, C, I, and S), based on a multilocus phylogeny. Genomes are still scarce, however, and it is unclear how the phylogenetic diversity, the broad ecological tolerances, and the ability to form symbioses with many different fungal host species are reflected in genome-wide differences. Here, we generated PacBio-based de novo genomes of six *Trebouxia* lineages belonging to the Clades A and S, isolated from lichen individuals of the genus *Umbilicaria*. Sequences belonging to Clade S have been reported in a previous study, but were reassembled and reanalyzed here. Genome sizes ranged between 63.08 and 73.88 Mb. Repeat content accounted for 9% to 16% of the genome sequences. Based on RNA evidence, we predicted 14,109 to 16,701 gene models per genome, of which 5,203 belonged to a core set of gene families shared by all 6 lineages. Between 121 and 454, gene families are specific to each lineage. About 53% of the genes could be functionally annotated. The presence of biosynthetic gene clusters (6 to 17 per genome) suggests that *Trebouxia* algae are able to synthesize alkaloids, saccharides, terpenes, NRPSs, and T3PKSs. Phylogenomic comparisons of the six strains indicate prevalent gene gain during *Trebouxia* evolution. Some of the gene families that exhibited significant evolutionary changes (i.e. gene expansion and contraction) are associated with metabolic processes linked to protein phosphorylation, which is known to have a role in photosynthesis regulation, particularly under changing light conditions. Overall, there is substantial genomic divergence within the algal genus *Trebouxia*, which may contribute to the genus’ large ecological amplitude concerning fungal host diversity and climatic niches.

**Significance**

The common lichen photobiont genus, *Trebouxia*, has a worldwide distribution and exhibits extensive phylogenetic and ecological diversity. However, genomic differences among lineages in this genera are currently unknown. We find extensive genomic divergence and an overall global pattern of gene expansion, which may be linked to lineage-specific environmental adaptation. Our study provides new insights into the genomic features and physiological potential of photobionts, which can contribute toward the understanding of the dynamics of the lichen symbiosis, and adaptive evolutionary processes of lichen populations.

## Introduction

Lichens are symbiotic associations primarily consisting of a fungal partner and one or more photosynthetic symbionts. Photobionts are unicellular green algae (Chlorophyta), or, less frequently, cyanobacteria. About 90% of the lichen-forming fungal species associate with green algal photobionts (Tschermak-Woess 1988). Among the green algal photobiont genera (e.g. *Trebouxia*, *Asterochloris*, *Trentepohlia*, and *Coccomyxa*), *Trebouxia* is the one most frequently encountered in lichen thalli (Ahmadjian 2001; Muggia et al. 2020). Lichens harboring *Trebouxia* symbionts occur in all terrestrial habitats, from the tropics (e.g. Kosecka et al. 2022) to Antarctica (e.g. Romeike et al. 2002).

Abiotic and biotic factors influence the occurrence of *Trebouxia* lineages. Climate variables, such as temperature and humidity, drive *Trebouxia* community composition along environmental gradients (Castillo and Beck 2012). Niche modeling approaches have revealed *Trebouxia* lineages that prefer particular climatic conditions (Rolshausen et al. 2018), or substrate pH (Peksa et al. 2022). While light availability is important for photosynthetic organisms like *Trebouxia*, excessive solar irradiance can, however, cause an adverse impact on its photosynthetic efficiency, thereby affecting the overall survival of a lichen holobiont (Casano et al. 2011; Miguez et al. 2017). In addition, the identity of the fungal host is an important factor influencing the distribution of *Trebouxia* lineages. Many species of lichenized fungi prefer certain *Trebouxia* strains (Leavitt et al. 2015), and pick up particular lineages selectively from *Trebouxia* communities present in the environment (Leavitt et al. 2015). Thus, fungal host identity, climate, and geographic factors work together in shaping *Trebouxia* distribution patterns (Singh et al. 2017; Dal Grande et al. 2018; Moya et al. 2021).

A single species of lichen-forming fungus can form associations with multiple *Trebouxia* lineages (e.g. Piercey-Normore 2006; Sadowska-Deś et al. 2013; Garrido-Benavent et al. 2023). It is thought that this flexibility toward the photobiont broadens the geographic range, and the ecological niche of the symbiotic consortium as a whole (Muggia et al. 2014; Rolshausen et al. 2020). Symbiont flexibility is often encountered in lichenized fungal species with large ranges, spanning multiple biomes, e.g. *Cetraria aculeata*, *Tephromela atra*, *Umbilicaria phaea*, and *Umbilicaria pustulata* (Muggia et al. 2010; Fernández-Mendoza et al. 2011; Rolshausen et al. 2020). Indeed, all of the aforementioned studies found evidence for unrelated *Trebouxia* strains with a preference for the Mediterranean climate zone, suggesting that these strains have independently evolved physiological adaptations to Mediterranean climatic conditions (hot and dry summers, wet winters, and no frost year-round). Multiple *Trebouxia* lineages may also be present in a single lichen individual (Dal Grande et al. 2014; Paul et al. 2018; Škaloud et al. 2018), and these lineages may have different physiological properties (Casano et al. 2011), suggesting they buffer the lichen consortium as a whole against variable environmental conditions.

*Trebouxia* is a phylogenetically diverse taxon; however, the vast majority of species-level lineages in *Trebouxia* are not formally described (Muggia et al. 2020). Establishing a phylogeny and an integrated taxonomy of the genus has been an ongoing endeavor by the scientific community for the past decade (Sadowska-Deś et al. 2014; Muggia et al. 2017, 2018, 2020; Nelsen et al. 2021). A recent multilocus phylogeny reports four major *Trebouxia* clades, A, C, I, and S, which are morphologically distinguished by pyrenoid structures (Muggia et al. 2020). The different clades occupy a wide range of habitats, from cool and dry high-altitude areas to warm and wet low-altitude habitats (Medeiros et al. 2021; Nelsen et al. 2021). Some studies have found that members of the different clades preferentially occupy a particular climate zone, e.g. members of Clade C tend to occupy warm and wet habitats, while members of Clades I and S occupy cool and dry habitats. On the other hand, members of Clade A can occupy two differing habitats (Nelsen et al. 2021). These findings imply that members of *Trebouxia* exhibit different environmental tolerances; however, it is unknown how these differences are reflected in their genomes.

The genomic resources currently available on the genus *Trebouxia* include a mitochondrial genome (Martinez-Alberola et al. 2020), a chloroplast genome (Martinez-Alberola et al. 2019), a nuclear genome based on Illumina reads (Kono et al. 2017), and a nuclear genome based on PacBio reads derived from a metagenomic sample (Greshake et al. 2020). Three genomes belonging to Clade S were reported in Puginier et al. (2024), and reassembled and reanalyzed here. Comparative genomics studies within *Trebouxia* including multiple strains are still lacking.

In this study, we generated genome assemblies from pure isolates of six *Trebouxia* strains to answer two specific questions: (i) How do different strains of *Trebouxia* vary in their genome compositions? and (ii) What are the gene families, core to *Trebouxia*, that underwent significant evolutionary changes?

## Results

### Genome Statistics and Taxonomic Placement

All generated and annotated genomes showed high BUSCO completeness (95.9% to 96.8%) and contiguity (N50 size ranging 0.531 to 2.89 Mb; Table 1). The different strains vary in genome sizes, ranging from 63.08 to 73.88 Mb, with guanine + cytosine (GC) content of 49.64% to 51.05%. Similarly, the total number of identified genes also varies among strains, which range from 14,109 to 16,701.

**Table 1:** Summary statistics of all generated and annotated *Trebouxia* genomes

Statistics	*Trebouxia* Clade S			*Trebouxia* Clade A		
	S09 C0004	S19 C0005	S12 C0006	A06 C0007	A10 C0009	A04 C0010
Genome size (Mb)	71.10	63.08	68.09	70.87	73.88	66.16
Number of contigs	170	289	60	60	88	78
Mean coverage	301.66×	242.09×	321.33×	223.63×	198.13×	377.08×
Largest contig (Mb)	4.40	2.03	3.66	3.90	4.72	3.85
N50 (Mb)	1.57	0.531	2.46	2.89	2.02	2.28
GC (%)	49.70	49.89	49.64	50.84	51.05	51.02
BUSCO Completeness (%)	91.1	91.3	91.3	90.8	91.2	91.0
	**96.8**	**95**.**9**	**96**.**5**	**96**.**6**	**96**.**3**	**96**.**4**
Complete and duplicated BUSCOs	1.4	0.9	0.7	0.7	0.9	0.5
	**1.3**	**1**.**0**	**1**.**0**	**0**.**9**	**1**.**0**	**0**.**9**
RNA-Seq mapping rates (%)	99.97	99.99	99.97	99.94	99.97	99.99
No. of genes	16,701	14,278	15,155	14,427	15,484	14,109
Gene density (genes/Mb)	235	226	223	204	210	213
No. of proteins	19,362	16,577	17,570	16,854	16,980	16,367
Unique proteins	5,164	3,337	4,173	3,643	4,100	3,596
Single-copy ortholog	3,399	3,399	3,399	3,399	3,399	3,399

BUSCO scores are shown for both genome assemblies and annotated gene sets (in bold).

Phylogenetic analysis of the *ITS2* locus showed that *Trebouxia* strains—C0004, C0005, and C0006—cluster with sequences previously identified as Clade S, while the strains C0007, C0009, and C0010 cluster with Clade A (see Supplementary material). The specific clade code for each *Trebouxia* strain is mentioned thereafter.

### Gene Families Core to *Trebouxia* and Evolutionary Changes

**Fig. 1.**
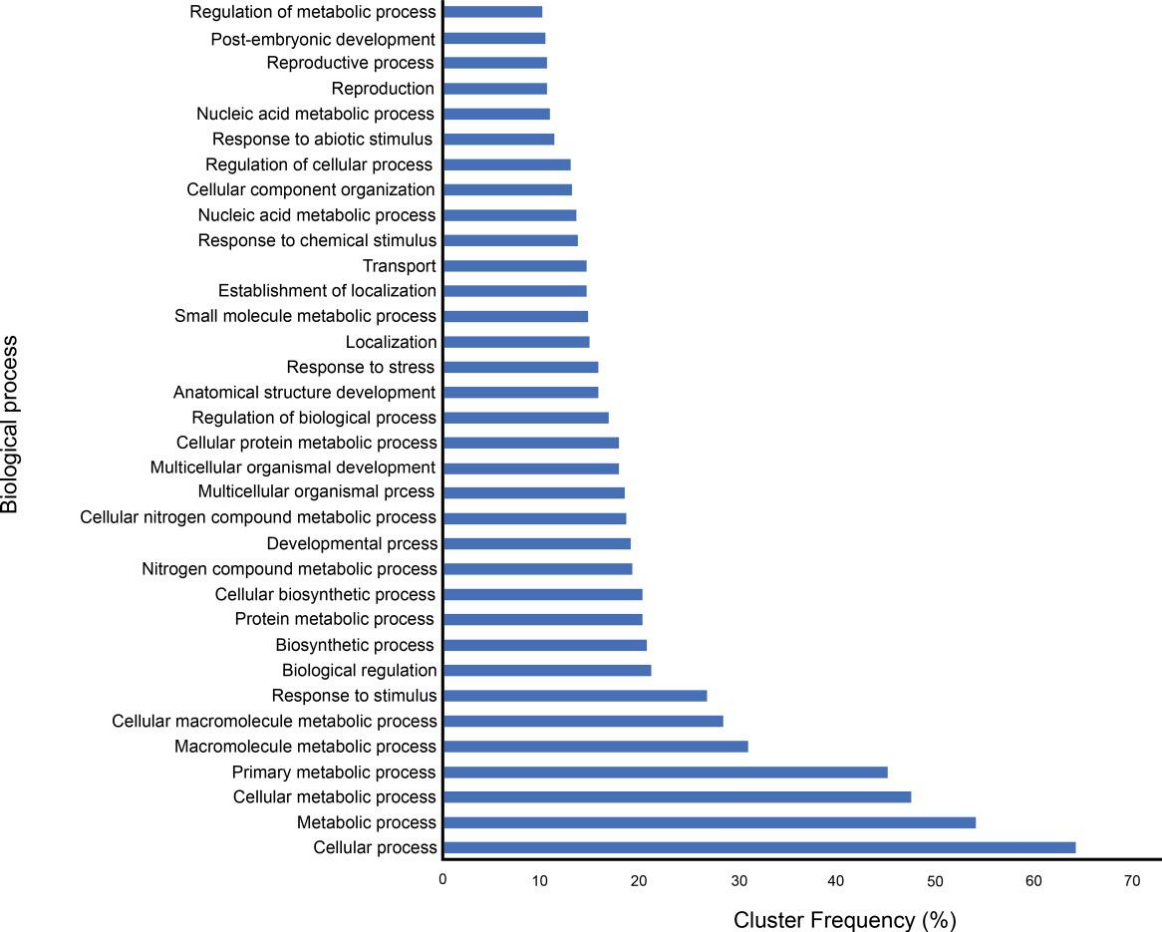
Results from GO enrichment analysis of gene families, core to *Trebouxia*, showing significantly enriched GOs (FDR ≤ 0.01) with corresponding cluster frequency (%; i.e. proportion of the number of genes annotated to a certain GO term to the overall number of genes with GO annotations). Only GO terms with at least 10% cluster frequency are shown here.

There are 5,203 gene families found common across the different strains of *Trebouxia* examined here (core gene families), in which between 121 and 454 gene families are specific to each lineage. Collinear genes can be found between closely related strains (supplementary fig. S1, Supplementary Material online). For strains belonging to Clade A, the percentages of shared collinear genes when compared between each other, range from 63.26% to 67.30%. On the other hand, the percentages of shared collinear genes between strains belonging to Clade S range from 56.50% to 66.91%. Analysis of overrepresentation of gene ontology (GO) categories (false discovery rate [FDR] ≤ 0.01) from gene family set core to *Trebouxia* showed signification enrichment of genes largely associated to cellular and metabolic processes (Fig. 1 and Supplementary material).

There were 490 core gene families (out of 5,203; supplementary table S2, Supplementary Material online), which showed significant evolutionary changes at *P* ≤ 0.05 using gamma distribution (highest likelihood at *k* = 2). Regardless of the specific gene family, there is a global pattern of more expansion (gains) than contraction (loss) for all genomes (Table 2). In particular, strains belonging to Clade S generally showed more gene gains than those belonging to Clade A. For Clade S, there were 423 and 299 gene families that showed significant expansions and contractions, respectively. On the other hand, there were 350 expansions and 287 contractions of gene families for strains belonging to Clade A.

**Table 2:** Total number of gene families that showed significant evolutionary changes (i.e. expanded and contracted; *P* ≤ 0.05) across different *Trebouxia* strains, with corresponding number of gene gains and losses

*Trebouxia* strain	Gene families		Genes	
	Expansion	Contraction	Gain	Loss
S09 C0004	160	66	359	78
S19 C0005	123	125	271	169
S12 C0006	140	108	318	132
A06 C0007	93	103	203	112
A10 C0009	100	113	228	137
A04 C0010	157	71	273	87

**Fig. 2.**
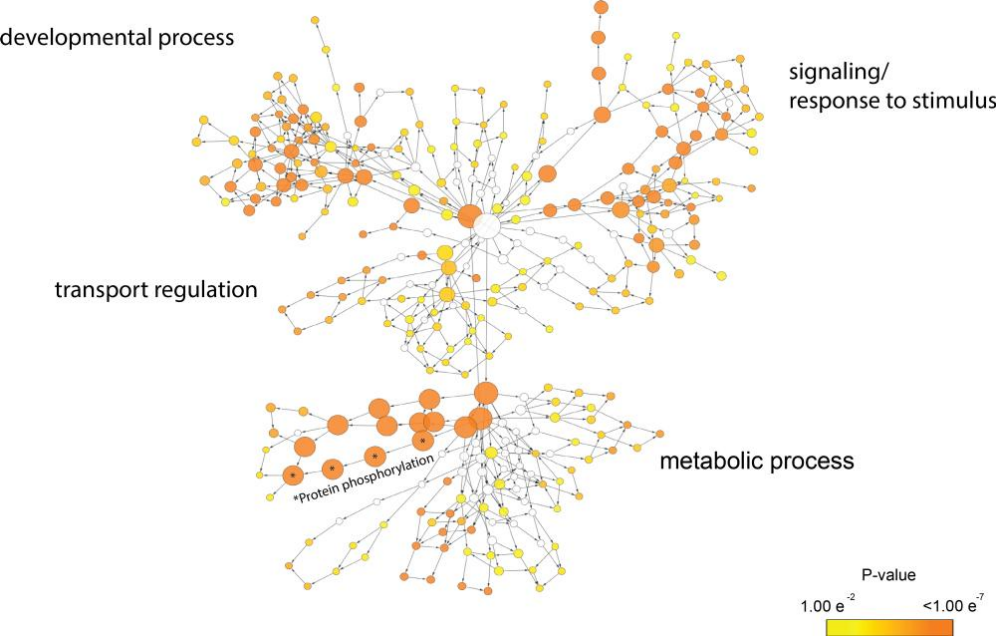
Results from GO enrichment analysis of gene families that underwent significant evolutionary changes, in which shaded nodes represent GO terms that showed significant overrepresentation (FDR ≤ 0.01). The node size is proportional to the number of gene families annotated to the corresponding GO term. Larger nodes with black asterisks represent highly overrepresented GO terms specifically linked to protein phosphorylation.

Functional profiling of gene families that underwent significant changes showed overrepresentation (FDR ≤ 0.01) of GO terms associated to developmental process, signaling and response to stimulus, transport regulation and metabolic process (Fig. 2). Further, there were a relatively high number of gene families annotated to GOs largely associated with metabolic process. Specifically, there was significant overrepresentation of processes specifically linked to phosphorylation (i.e. 120 out of 490 core gene families that underwent significant changes) such as genes encoding for protein kinase and phosphatase (Fig. 2 and Table 3). The direction of the evolutionary pattern (i.e. gain or loss), however, varies depending on the strain.

**Table 3:** Evolutionary changes of core *Trebouxia* gene families associated to phosphorylation with known protein matches, showing number of significant gene gains (+) and losses (−), as well as no significant change (0) found for respective *Trebouxia* strains

Gene family	Protein name	S09_C0004	S19_C0005	S12_C0006	A06_C0007	A10_C0009	A04_C0010
OG0000088	LRR receptor-like serine/threonine-protein kinase	+5	−5	1	1	−1	0
OG0000217	Baculoviral IAP repeat-containing protein 6	+4	−1	0	0	−1	0
OG0000218	Probable serine/threonine-protein kinase PkwA	+5	0	−1	−1	0	0
OG0000219	Serine/threonine-protein kinase Constitutive Triple Response 1	−1	0	0	+3	−3	+1
OG0000238	LRR receptor-like serine/threonine-protein kinase GASSHO 1	+2	0	0	0	−1	0
OG0000240	Serine/threonine-protein kinase RUNKEL	0	−1	+3	0	0	0
OG0000242	Chitin elicitor receptor kinase 1	+4	0	0	+1	0	0
OG0000275	Phosphatidylinositol-3-phosphatase SAC1	+4	0	−1	−1	0	0
OG0000393	Casein kinase 1-like protein HD16	0	−1	0	+2	−2	0
OG0000417	Cysteine-rich receptor-like protein kinase 41	+3	0	0	+1	0	0
OG0000536	Casein kinase I isoform beta	0	+2	0	+1	+1	0
OG0000551	U-box domain-containing protein 34	+1	0	−1	−1	0	0
OG0000636	Uncharacterized sugar kinase	0	+2	−1	0	−1	0
OG0000652	Serine/threonine-protein kinase Nek1	+2	0	−1	0	−1	0
OG0000713	Glycerol kinase 3	+2	−1	0	−1	0	0
OG0000746	Polyribonucleotide 5″-hydroxyl-kinase Clp1 (EC 2.7.1.78)	+2	0	0	0	0	+4
OG0000895	*N*-acetylmuramic acid/*N*-acetylglucosamine kinase	0	0	+1	0	−1	+2
OG0000914	Putative GTP diphosphokinase RSH1, chloroplastic	0	+3	−1	0	0	0
OG0000936	Thiamine biosynthetic bifunctional enzyme BTH1, chloroplastic	0	+1	−1	0	0	+2
OG0000958	Osmotic stress/abscisic acid-activated protein kinase 2	+1	−1	+1	0	+1	0
OG0000962	Casein kinase II subunit alpha-2	0	+1	0	+1	−1	+1
OG0000988	AarF domain-containing protein kinase 1	0	+1	0	0	0	+4
OG0001248	[3-Methyl-2-oxobutanoate dehydrogenase [lipoamide]] kinase, mitochondrial	0	+2	0	0	+2	0
OG0001333	Protein phosphatase 2C 57, Thylakoid-associated phosphatase of 38 kDa	+1	0	0	0	0	+3
OG0001335	Myristoylated and palmitoylated serine/threonine-protein kinase	0	+2	0	0	+2	0
OG0001518	Probable choline kinase 2	+1	0	0	0	0	+3
OG0002057	Probable protein phosphatase 2C 26	0	0	+3	0	0	0

### Genome Divergence Within *Trebouxia*

**Fig. 3.**
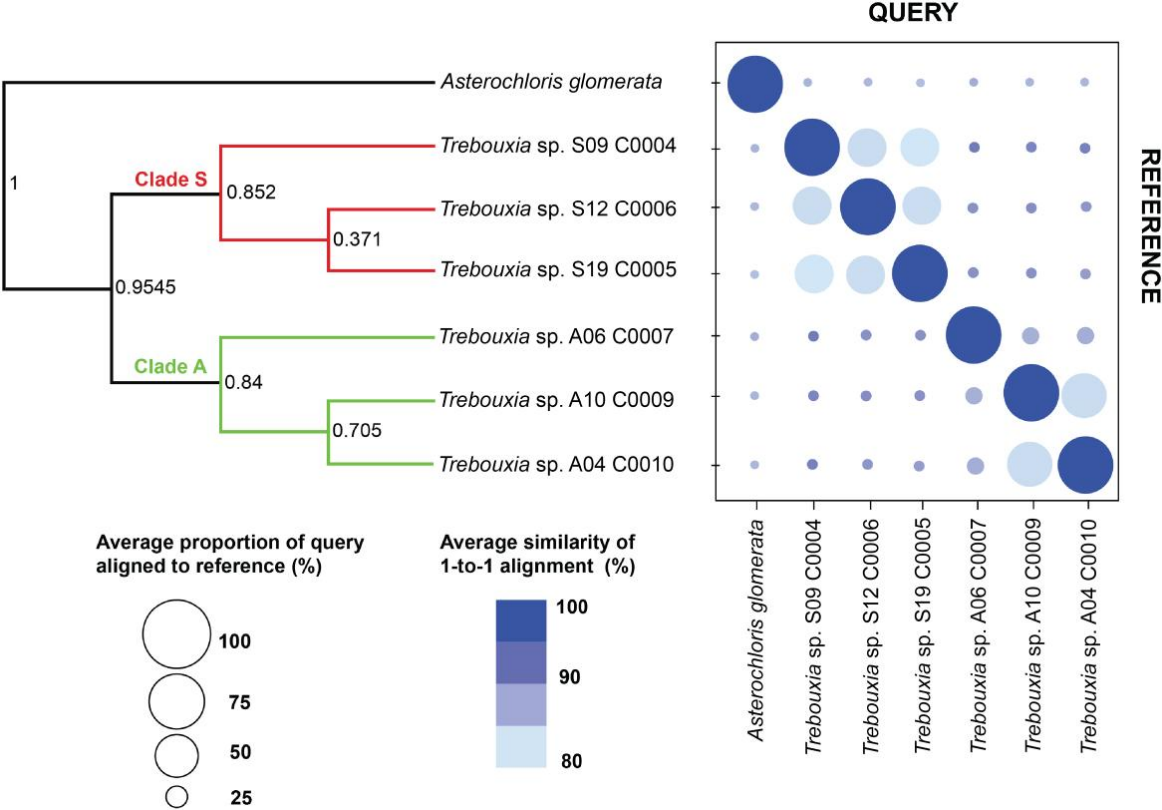
Pairwise comparisons between the genomes of *Trebouxia* generated here and an outgroup species—*A. glomerata*. The size of the circle is proportional to the percentage of the genome that aligned between a pair of genomes and the color of the circle depicts the average percent identity of the best reciprocal one-to-one aligned regions. The larger and darker the circles are, the higher the similarity between a genome pair. The phylogeny is based on whole-genome data. Branch support values were derived from single-locus gene trees supporting each bipartition as a measure of support via the STAG algorithm implemented in the in Orthofinder.

Results from pairwise comparisons showed genome sequences of an outgroup species, *Asterochloris glomerata*, and those belonging to the genus, *Trebouxia* are very divergent (*Q* = 0.04%; *I* = 87.16% to 88.29%; Fig. 3). Remarkably, genomes within *Trebouxia* also showed extensive divergence, for instance, comparisons between genome pairs S12_C0006 and A06_C0007 (*Q* = 1.35% and *I* = 90.37%), as well as S09_C0004 and A10_C0009 (*Q* = 1.55% and *I* = 91.12%), respectively. As expected, genomes of the same clades generally showed relatively high sequence similarities: *Q* = 45.71% to 56.57% and *I* = 83.37% to 85.91% for genome pair comparisons within Clade S. Similarly, the genomes for A10_C0009 and A04_C0010, both from Clade A, also showed high similarities with *Q* = 64.41% and *I* = 85.93%. However, within-clade divergence was also observed for A06_C0007 in comparisons with A10_C0009 and A04_C0010: *Q* = 13.36% to 14.45% and *I* = 88.48% to 88.68%.

### Genome Repeat Composition

**Fig. 4.**
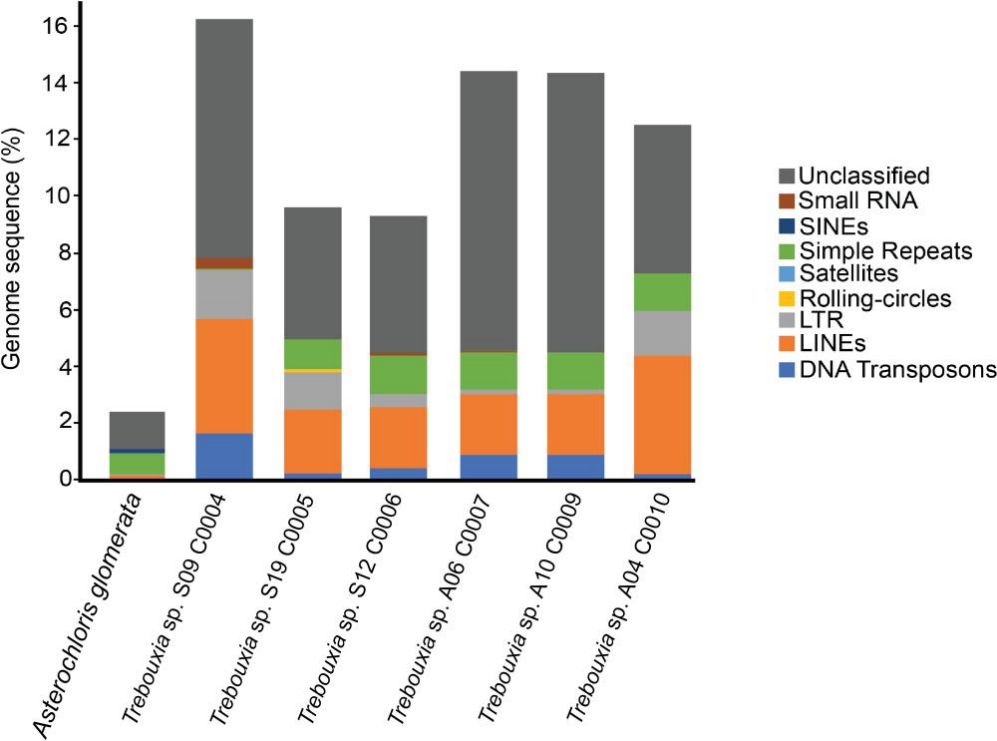
Repeat composition found in the genomes of the six *Trebouxia* strains and *A. glomerata*.

The contribution of repeats to the genomes of the six *Trebouxia* strains is higher (9.34% to 16.32%; Fig. 4) than in *A. glomerata* (2.36%). The genomes of *Trebouxia* contain predominantly unclassified repeats and transposable elements (TEs), particularly LINE (Fig. 4). However, the distribution of LINE subtypes varies depending on the strain (supplementary fig. S2, Supplementary Material online). For instance, the LINE superfamilies Jockey-I and CR1 were only found for *Trebouxia* S19 C0005 and A06 C0007, respectively. Kimura distances (*K* values) of TEs elements showed both highly divergent ancient copies (high *K* values) and less divergent recent copies (low *K* values), which generally involved LINE, and to some extent long terminal repeat (LTR) and DNA transposons, depending on the strain.

### Biosynthetic Gene Clusters

**Fig. 5.**
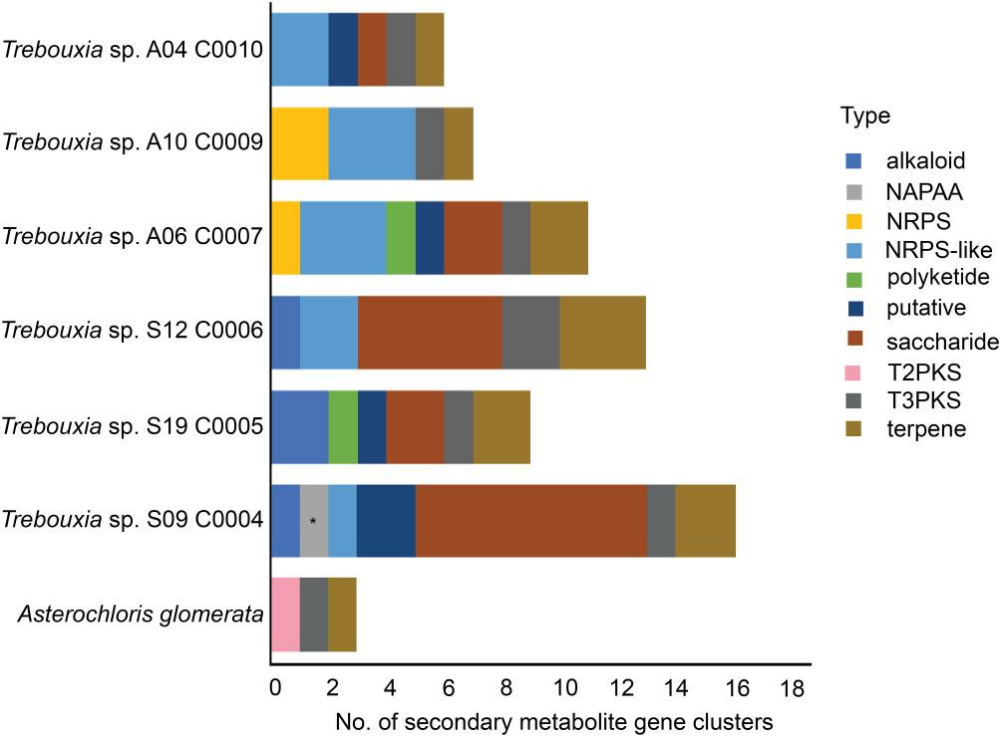
Different types of BGCs found in the genomes of the six *Trebouxia* strains and the outgroup species, *A. glomerata*. The category type “putative” represents clusters that are putatively BGCs but no known types found in the antismash database. Bar indicated with asterisk depicts BGCs that showed similarity match to known compounds—NAPAA (100% match to ε-poly-L-lysine).

Overall, the *Trebouxia* genomes had a higher number of biosynthetic gene clusters (BGCs) than *A. glomerata* (3). The strain A04 C0004 had a particularly high number of BGCs (17) compared with other strains—S19 C0005 (9); S12 C0006 (13); A06 C0007 (11); A10 C0009 (7); and A04 C0010 (6) (Fig. 5). All of the strains contained clusters linked to terpene and Type III PKS biosynthesis. The majority of the algal strains also contained NRPS/NRPS-like gene and saccharide clusters, except for S19 C0005 and A10 C0009, respectively. An uncategorized polyketide was also identified, but found only in strains S19 C0005 and A06 C0007.

## Discussion

The most common lichen photobiont genus, *Trebouxia*, is known to exhibit a wide range of environmental tolerances; however, knowledge about the genomic makeup of this taxon is still scarce. Here, we generated high-quality genome assemblies from six strains of *Trebouxia*, and performed a series of comparative genomic analyses to obtain the first estimate of genome content variation within the genus. Our findings show extensive genomic variation within this lichen photobiont genus, and even among strains of the same clade. We also report gene families, core to *Trebouxia* examined here, that underwent significant evolutionary changes in which the global pattern shows more gene expansion than contraction.

The genomic features of the different *Trebouxia* strains vary in a number of aspects including the genome size, number of contigs, N50 length of contig, and number of protein-coding genes (Table 1). Highly accurate HiFi reads generated from PacBio circular consensus sequencing (CCS) sequencing resulted in more contiguous genome assemblies and larger N50 length of contigs for the assembled genomes of strains A06 C0007, A10 C0009, and A04 C0010. On the other hand, the genome assemblies for strains that were sequenced in the PacBio continuous long read (CLR) platform were relatively less contiguous and shorter N50 of contigs (i.e. S09 C0004 and S19 C0005), except for S12 C0006.

### Genome Size and Genome Divergence

The sizes of the genomes generated here are within the range of expected genome sizes based on publicly available genomes (Kono et al. 2017; Greshake et al. 2020). The genomes generated here are, however, relatively more complete (BUSCO ∼96%, based on Chlorophyta) than the published genome of *Trebouxia* isolated from *Usnea hakonensis* (BUSCO 91%).

Pairwise comparisons of all possible combinations of the six *Trebouxia* strains and the outgroup species, *A. glomerata*, showed extensive divergence, not just within the genus but also within clade (Fig. 2). As expected, strains that belong to the same clade showed higher similarities than strains belonging to different clades. However, there is substantial genomic variation even between strains within the same clade (i.e. Clade A), in which the genome of A06 C0007 appears different from those of A10 C0009 and A04 C0010.

### Gene Prediction Genome Annotation and Functional Profiling

Green algal genomes have variable numbers of protein-coding genes, ranging from 7,500 to 18,000 (Foflonker and Blaby-Haas 2021). The number of gene models found in *Trebouxia* in the present study is comparable with other single-celled green algae, such as members of the genus *Chlamydomonas* with 15,500 to 16,600 genes (Craig et al. 2021) or the lichen-associated *Coccomyxa viridis* with 13,500 predicted protein-coding genes (Kraege et al. 2023). Interestingly, the strain S09 C0004 contains a much higher number of predicted genes than the other five analyzed genomes, despite having a similar assembly size (Table 1), indicating that this species has a higher gene density.

Typically, only a small amount of the predicted genes in green algal genomes can be annotated (Foflonker and Blaby-Haas 2021). In our analyses, ∼53% of the predicted genes could be annotated. Considering algae being a poorly characterized group and nonmodel organisms, the low percentage of annotation is likely due to the method of annotation that is generally based on sequence similarity searches (Blaby-Haas and Merchant 2019). Further, the results from functional profiling of core *Trebouxia* genes showed significant enrichment of GOs largely associated with metabolic processes (Fig. 1), which can be attributed to them being primary producers and their capacity to produce metabolites (Park et al. 2022; Pichler et al. 2023). Phototrophic eukaryotes, including plants and algae, are known to produce both primary (e.g. lipids) and secondary metabolites (e.g. carotenoids; Park et al. 2022), which support our findings showing BGCs in the genomes of the six *Trebouxia* strains. The number of BGCs found in the genomes, however, varies depending on the strain, suggesting differences in their environmental tolerances considering the role of natural compounds in the organism–environment interaction (reviewed in Huneck 1999).

### Genome Repeat Composition

The majority of eukaryotes contain repetitive elements in their genomes, including mobile elements (i.e. TEs), which can drive genetic variability, thereby influencing evolutionary trajectories (van de Lagemaat et al. 2003; Lopes et al. 2008). Here, we found a range of 3.04% to 7.4% (Fig. 3) TEs in the genomes of *Trebouxia*, which is comparable with the well-studied green algal species, *Chlamydomonas reinhardtii*, which contained about 6% to 7% TEs in its genome (Philippsen et al. 2016). Whether these TEs significantly contributed to differences in genome size among *Trebouxia* still remains unclear considering they are present at only low levels. While TEs found in the analyzed *Trebouxia* genomes are predominantly LINEs, the subtypes are strain specific. Thus, this variation potentially contributed to the genomic variability observed among *Trebouxia* strains.

### Biosynthetic Gene Clusters

Microalgae are valued for their capacity to synthesize natural products (Michalak and Chojnacka 2015; Sathasivam and Ki 2018; Tang et al. 2020). Mining the genomes of microalgae for biosynthetic genes can provide an estimate of the biosynthetic potential of these green algal strains (Gulshan et al. 2020). Biosynthetic genes previously reported from the genus *Trebouxia*—based on metagenomic sequencing of entire lichen thalli—include genes linked to the biosynthesis of terpenes, NRPSs, and Type III PKSs (Ahmad et al. 2023). Our study confirms that these classes of BGCs are commonly found in members of *Trebouxia*. In addition, we show that BGCs associated with saccharide biosynthesis are present in most *Trebouxia* genomes, in variable numbers (1 to 8). The potential to synthesize alkaloids appears to be clade specific, and limited to members of Clade S of *Trebouxia* (Fig. 3). Among all six strains examined here, only S09 C0004 showed a similarity match of a BGC to a known compound, specifically to the antimicrobial compound ε-poly-L-lysine (100% similarity). A recent review of algal metabolites by Bhowmick et al. (2020) showed a wide range of primary and secondary metabolites synthesized by different algae from freshwater and marine environments, with extensive antibacterial activity against pathogenic bacteria. Hence, synthesis of antimicrobial compounds, such as ε-poly-L-lysine, in *Trebouxia* is possible.

### Evolutionary Changes in Core *Trebouxia* Gene Families

Findings from studies that examine expansions and contractions in gene families of related taxa can contribute to our understanding of evolutionary trajectories of specific traits (Hahn et al. 2005; Emms and Kelly 2019). Changes in copy number can represent an adaptive mechanism such that more copies of a specific gene (i.e. expansion) increase production of the corresponding protein, thereby allowing the organism to better deal with some tasks which in turn can increase its overall fitness (Hastings et al. 2009; Freitas and Nery 2020). Nevertheless, both expansion and contraction of gene families can indicate adaptation of an organism to a particular lifestyle (Sharma et al. 2018; Freitas and Nery 2020).

Our findings showed a global pattern of more expansion (gains) than contraction (loss) for all six *Trebouxia* strains. We found significant changes and highly enriched GOs associated to metabolic processes specifically linked to phosphorylation. Interestingly, there are strain-specific evolutionary changes (i.e. expansion or contraction) of gene families encoding for protein kinase and phosphatase (Table 3). Interestingly, the strain S09 C0004 exhibits a relatively higher number of gene gains for different protein kinases compared with other strains, which can potentially manifest as high acclimatization capacity to changing conditions. We observed this particular strain associated with *U. pustulata* living in varying elevations and thus, differing climate conditions. In photosynthetic organisms such as algae, it is known that protein phosphorylation plays a critical role in mediating the photosystems under changing light conditions. In particular, protein kinases have been reported as critical regulators of the core proteins in Photosystems II (PSII), including D1, D2, CP43, and PsbH (Rintamäki et al. 1997; Rochaix et al. 2012). Damage repair cycle of the PSII in response to changing environmental conditions require a series of phosphorylation and dephosphorylation of the core PSII proteins (Rintamäki et al. 1997; Vener et al. 2001).

## Conclusion

We find substantial genomic variation within the large and ecologically diverse lichen photobiont genus, *Trebouxia*. It remains to be shown, in how far the observed variation within the genus contributes to *Trebouxia*'s large ecological amplitude. Importantly, the genome assemblies generated in this study provide genomic resources for future studies looking into specific genes associated with a particular algal trait (e.g. photosynthetic efficiency), which can refine our understanding of how different strains cope with different ecological conditions. Given the essential role of the photosynthetic partner in a lichen symbiosis, a better understanding of the genomic features and physiological potential of photobionts can overall provide insights into the dynamics of the symbiosis, and adaptive evolutionary processes of lichen populations.

## Materials and Methods

### Algal Cultures

Pure isolates of six *Trebouxia* strains were used in this study to generate genome assemblies. These strains were previously isolated from the lichen thalli of two *Umbilicaria* species—*U. phaea* (strains: C0007, C0009, and C0010) and *U. pustulata* (strains: C0004, C0005, and C0006)—collected at low and high elevation sites in the United States of America (California) and Europe (Spain), respectively (supplementary table S1, Supplementary Material online). The photobionts were isolated using a single-cell micromanipulator following the procedures described in Beck and Koop (2001) and grown on solid Bold's Basal Medium with vitamins and triple nitrate (Ahmadjian 1969). The algal cultures are maintained in a growth chamber under 60 μmol/m^2^/s photosynthetic photon flux density with a 12-hour photoperiod at 16 °C.

### Nucleic Acid Isolation

Dry samples from pure cultures of *Trebouxia* algae isolated from *U. phaea* were flash-frozen in liquid nitrogen, and ground to a fine powder in preparation for genomic DNA (gDNA) and RNA isolation procedures (supplementary fig. S3, Supplementary Material online). The homogenized algal material (∼500 mg) was transferred into 10 ml sodium dodecyl sulfate (SDS)-based lysis buffer (100 mM Tris pH 8.0, 0.5 M NaCl, 50 mM ethylenediaminetetraacetic acid, 1.25% SDS, 1% sodium metabisulfite, 1% polyvinylpyrrolidone 30, 1% 2-mercaptoethanol) preheated to 65 °C (Mayjonade et al. 2016). The mix was incubated at 65 °C for 15 min, then 50 µl RNAse A (20 mg/ml) was added to ensure the complete removal of RNA from the sample. After further incubation for 60 min at 65 °C, one volume of chloroform: isoamyl alcohol (24:1, v/v) was added and mixed by inversion for 5 min and spun at 10,000 × *g* for 10 min at 4 °C. To precipitate the proteins and polysaccharides the upper aqueous layer was pipetted into a new tube and 0.3 volumes of 5 M potassium acetate were added. The resulting mix was incubated for 5 min on ice and centrifuged at 8,000 × *g* for 10 min at 4 °C. The supernatant was transferred to a new 50 ml tube, one volume of phenol: chloroform: isoamyl alcohol (25:24:1, v/v/v) was added and inverted for 5 min. After centrifugation at 10,000 × *g* for 10 min at 4 °C, further chloroform:isoamyl alcohol extractions were carried out until no interphase between the organic and aqueous phase could be detected. Afterwards, the gDNA was precipitated by adding 0.1 volume 3 M sodium acetate (pH 5.2) and 2.5 volumes of ethanol, followed by an overnight incubation at −20 °C. Thereafter, gDNA was pelleted by centrifugation at 12,000 × *g* for 30 to 60 min at 4 °C, washed with 70% ethanol and air-dried. Subsequently, the gDNA pellet was resuspended in molecular-grade water. Due to the high content of polyphenols, polysaccharides, and pigments, further purifications were carried out using the DNeasy PowerClean Cleanup Kit (Qiagen). The purifications were repeated until a 260/280 absorbance ratio of 1.75 to 1.85 and a 260/230 absorbance ratio of 2.0 to 2.2 were obtained. Quality of the gDNA samples was assessed with Nanophotometer (Implen, NanoPhotometer Pearl), Qubit 2.0 Flurometer (Thermo Fisher, using Qubit dsDNA high-sensitive [HS] assay) and 2,200 TapeStation (Agilent Technologies).

Isolation of RNA was performed using Quick-RNA Fungal/Bacterial Miniprep Kit (Zymo Research) with starting algal material of 30 to 50 mg. RNAs were further purified, when necessary, with the RNA Clean & Concentrator-5 Kit (Zymo Research). Total RNAs were sent to Novogene (Cambridge, England) for library preparation and mRNA sequencing on the Illumina NovaSeq platform (paired-end 150 bp sequencing read length).

### Library Preparation and Sequencing

For samples that passed quality control, SMRTbell libraries were constructed according to the manufacturer's instructions of SMRTbell Express Template Prep Kit 2.0 for low DNA input protocol (Pacific Biosciences, Menlo Park, CA, USA). Briefly, gDNA (250 to 900) was sheared to 20 kb 370 fragments using Megaruptor 2 (Diagenode, Belgium) and then bead-size selected with AMPure PB beads (Pacific Biosciences) to remove <3 kb SMRTbell templates. SMRT sequencing was performed on the Sequel System II platform, in CCS mode and 30 h movie time with preextension at the Genome Technology Center (RGTC) of the Radboud University Medical Center (Nijmegen, The Netherlands).

### Genome Assembly and Quality Assessment

The obtained PacBio raw data sequences for *Trebouxia* strains—C0007 and C0010—are in the format of “subreads.bam” while data for C0009 is in “reads.bam.” Thus, HiFi reads were extracted in two ways: (i) using *pbccs* (Version 6.4.0) with default parameters for subreads.bam and (ii) using a custom perl script for reads.bam. On the other hand, PacBio raw subreads for *Trebouxia* isolated from *U. pustulata* (i.e. C0004, C0005, and C0006), which were sequenced in CLR mode, were obtained from Puginier et al. (2024). Genome of each *Trebouxia* strain were then assembled, de novo, using Flye (Version 2.9), indicating the parameter −*pacbio-hifi* for HiFi reads and *−pacbio-raw* for CLRs.

The qualities of the generated genome assemblies were evaluated using BUSCO (Version 5.2.2; Manni et al. 2021a, 2021b) with default parameters and indicating the lineage Chlorophyta as the reference database (i.e. chlorophyta_odb10). In addition, quality statistics for each genome assembly was generated using backmap (Version 0.5; Schell et al. 2017; Pfenninger et al. 2022) which utilizes a number of built-in tools, including samtools, bwa mem, minimap2, qualimap, MultiQC, bedtools, and RScript (Li et al. 2009; Quinlan and Hall 2010; Li 2013; Ewels et al. 2016; Okonechnikov et al. 2016; Li 2018; Team 2021).

Putative contaminants were examined using MEGAN (Version 6.19) which performed taxonomic binning through sequence similarity matches. Visualization of GC content of contigs against sequence coverage for taxonomic partitioning was also performed using BlobTools (Version 1.1.1). However, removal of contigs which were not binned under the Phylum Chlorophyta in MEGAN and those with nontarget taxonomic matches in BlobTools caused significant reduction (∼30%) of BUSCO completeness. Additionally, we screened the genomes using the NCBI Foreign Contamination Screen (Version 0.5.0) and removed contigs that were flagged with contamination errors (i.e. non-*Trebouxia* match).

### Identification of *Trebouxia* Clade

Sequences of the internal transcribed spacer region 2 (*ITS2*) were extracted from all six *Trebouxia* genome assemblies generated here using ITSx (Version 1.13), indicating “Chlorophyta” to restrict the search to this lineage. The *ITS2* sequences extracted from ITSx were then used for multiple alignment with previously Sanger-sequenced *ITS2* from the same *Trebouxia* strains used here, together with published *Trebouxia ITS2* sequences (Leavitt et al. 2015; Muggia et al. 2020; Medeiros et al. 2021; Kosecka et al. 2022) using Geneious (Version 11.0.14.1 + 1) alignment tool with default parameters (global alignment with free end gaps, cost matrix: 65% similarity, gap open penalty: 12, gap extension penalty: 3, and refinement iterations: 2). A pairwise distance analysis was performed using the multiple alignment output, with the default Tamura-Nei Genetic Distance Model and Neighbour–Joining method without an outgroup. Branch supports were assessed via 100 replicates of bootstrap analysis.

### Genome Annotation

Annotation of genome assemblies was performed following the Funannotate (Version 1.8.13) pipeline (Palmer and Stajich 2019). Briefly, each genome was soft masked to identify some inherent repetitive elements. Initial training was performed using the RNA-seq data, which initiated a genome-guided Trinity RNA-seq assembly followed by PASA assembly. The training step generated alignments, Trinity transcripts, and PASA GFF3 annotation files which aided the next step. Gene prediction was subsequently performed by running *funannotate predict* which parsed the data provided from the training step and selected the best method to train the ab initio gene predictors (i.e. Augustus and GeneMark). Consensus of gene models from all the data provided was then generated with Evidence Modeler. Since RNA-Seq data was used in the training step, the command *funannotate update* was performed to add untranslated regions data to the gene prediction results and fixed gene models that were in disagreement with RNA-seq data. To aid the functional annotation, the built-in tools Eggnog mapper and InterProScan were performed. Additionally, prediction of transmembrane topology and signal peptides, as well as identification of BGCs were performed using phobius (https://phobius.sbc.su.se; accessed on 2023 February 25) and bacterial/fungal/plant versions of antiSMASH (Version 7.0; https://antismash.secondarymetabolites.org, https://fungismash.secondarymetabolites.org, http://plantismash.secondarymetabolites.org, accessed on 2023 April 19), respectively. To incorporate all the functional data generated above, the command *funannotate annotate* was performed. Finally, the annotated genomes were compared with each other with the command *funannotate compare* to perform descriptive summary statistics. BUSCO assessment of the annotated genomes was performed following the same procedures described above.

### Genome Sequence Divergence and Repeat Composition

Pairwise alignments of whole-genome sequences for all possible genome pair combinations were performed with mummer (Version 4.0.0b2) using the function *nucmer*. This tool evaluates sequence similarity between a pair of genomes based on the proportion of the total bases in the query genome that aligned to the reference genome (Q), as well as the average percent identity of the best reciprocal one-to-one aligned regions (I). Here, we included the genome data of a closely related lichen-forming algal species—*A. glomerata*—as an outgroup (Armaleo et al. 2019).

Further, repeat content for each genome was determined to examine whether there are any differences in proportion of repeats across different *Trebouxia* strains and the outgroup species, *A. glomerata*. A library of repeats was first created for each assembly using RepeatModeler (Version 2.0.1) and all repeats were masked using RepeatMasker (Version 4.1.0). Annotation of repeat types for each genome was manually curated to determine the proportion of each type. To assess copy divergence, the perl script *calcDivergenceFromAlign.pl* provided by the RepeatMasker tool was performed. This step generated Kimura distances (Kimura 1980) for the major types of TEs present in each genome, including long/short interspersed nuclear elements (LINES/SINES), LTR, and DNA transposons. Repeat landscape for each genome was created thereafter by running *createRepeatLandscape.pl* which generated graphs containing proportions of TE classes and the corresponding Kimura substitution level.

### Functional Analysis of Gene Families Core to *Trebouxia* and Evolutionary Changes

Protein sequences from all genomes were used to infer groups of homologous sequences (i.e. orthogroups) using OrthoFinder v2.5.4 (Emms and Kelly 2019) and each orthogroup is considered a gene family. The gene families that were found common (i.e. core) for all six *Trebouxia* strains were manually curated. Protein sequences of the core gene families were matched against the Uniprot database (downloaded on 2023 April 27) using ncbi-BLAST (Version 2.14.0) function *blastp*. Protein matches were then used for Functional annotation using the Uniprot Knowledgebase (https://www.uniprot.org/, accessed on 2023 May 15). Subsequently, enrichment analysis of GO terms was performed using the Biological Networks Gene Ontology tool (BiNGO V 3.0.5; Maere et al. 2005), available in Cytoscape (V 3.9.1, http://www.cytoscape.org/; Shannon et al. 2003; Maere et al. 2005), by running Hypergeometric test with Benjamin and Hochberg FDR multiple testing correction (significance level = 0.01) on the set of genes from the *blastp* output.

Change in gene family size was estimated using the software CAFE5 (Computational Analysis of Gene Family Evolution, Version 5.10; Hahn et al. 2005; De Bie et al. 2006; Mendes et al. 2021). This tool implements a birth–death model to infer gene family evolution and can perform computations that implement among family rate variation using a discrete gamma model by passing the “−k” parameter that indicates the number of rate categories to use. To aid in running the model, the output from Orthofinder was used, including a species tree containing the six *Trebouxia* strains and the outgroup species (i.e. *A. glomerata*), as well as the count data for each gene family. The species tree was generated using the STAG algorithm and rooted using the STRIDE algorithm. The STAG algorithm uses the proportion of species trees derived from single-locus gene trees supporting each bipartition as a measure of support (Emms and Kelly 2019).

As large gene copy number variance can cause unreliable parameter estimates, gene families that have one or more species with ≥ 100 gene copies were removed prior to running the model (Mendes et al. 2021). In total, 14,350 gene families were used to run the analysis. Multiple runs were performed, varying the parameter −k each time, until the highest likelihood value was achieved. Gene families that showed significant evolutionary changes (*P* ≤ 0.05), were manually curated. The pattern of changes for each gene family was retrieved from the output file generated from Cafe5 (i.e. Gamma_change.tab) in which values with plus (+) and minus (−) represent expansions and contractions, respectively. The corresponding numbers of gene gains and losses are based on the reconstructed count of genes from Cafe5 output (i.e. Gamma_count.tab). Subsequently, GO enrichment analysis was performed following the same procedure described above.

## Supplementary Material

evae219_Supplementary_Data

## Data Availability

The raw PacBio sequence reads and assembled genomes have been deposited in NCBI under the BioProject Accession numbers PRJNA790449 and PRJNA932175. The corresponding annotation for each genome and supplementary files are also available at: https://doi.org/10.5281/zenodo.10522906.
