# Supplementary material for: Genome-Wide Comparisons Reveal Extensive Divergence Within the Lichen Photobiont Genus, Trebouxia
Source: Genome Biol Evol. 2024 Oct 30;16(10):evae219. doi: 10.1093/gbe/evae219 (PMC11523091; doi:10.1093/gbe/evae219)
Supplement: evae219_Supplementary_Data [file evae219_supplementary_data.docx]

**Supplementary Table 1** General information on the different *Trebouxia* algal strains examined in this study.

| ***Trebouxia* strain** | **Fungal host species** | **Sampling Location** | **Climate zone** |
| --- | --- | --- | --- |
| S09_C0004 | *Umbilicaria pustulata* | Spain | Low and/or High |
| S19_C0005 | *U. pustulata* | Spain | Low |
| S12_C0006 | *U. pustulata* | Spain | High |
| A06_C0007 | *U. phaea* | California | Low |
| A10_C0009 | *U. phaea* | California | High |
| A04_C00010 | *U. phaea* | California | Low |


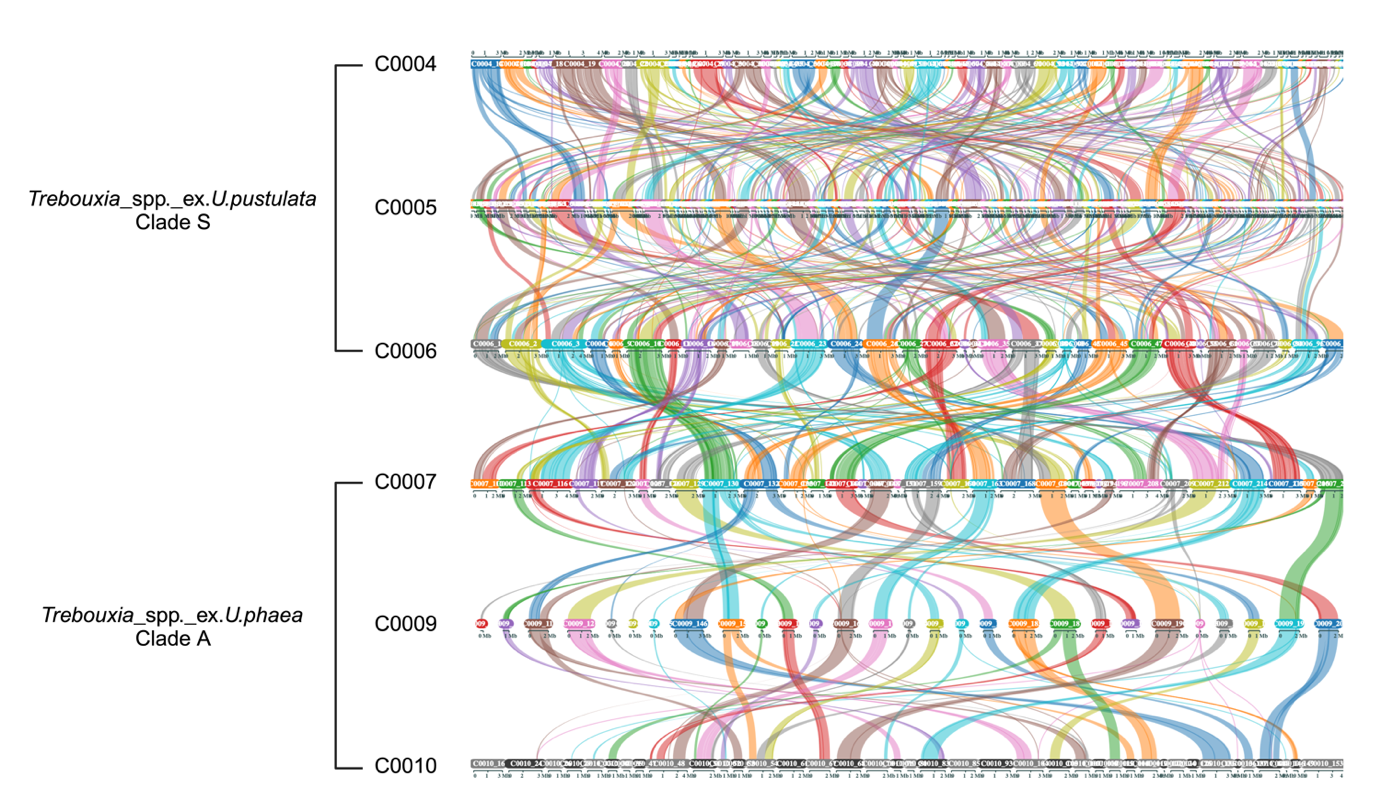


**Supplementary Figure 1** Synteny plot showing shared collinear genes among- and within the two Trebouxia clades examined in this study.


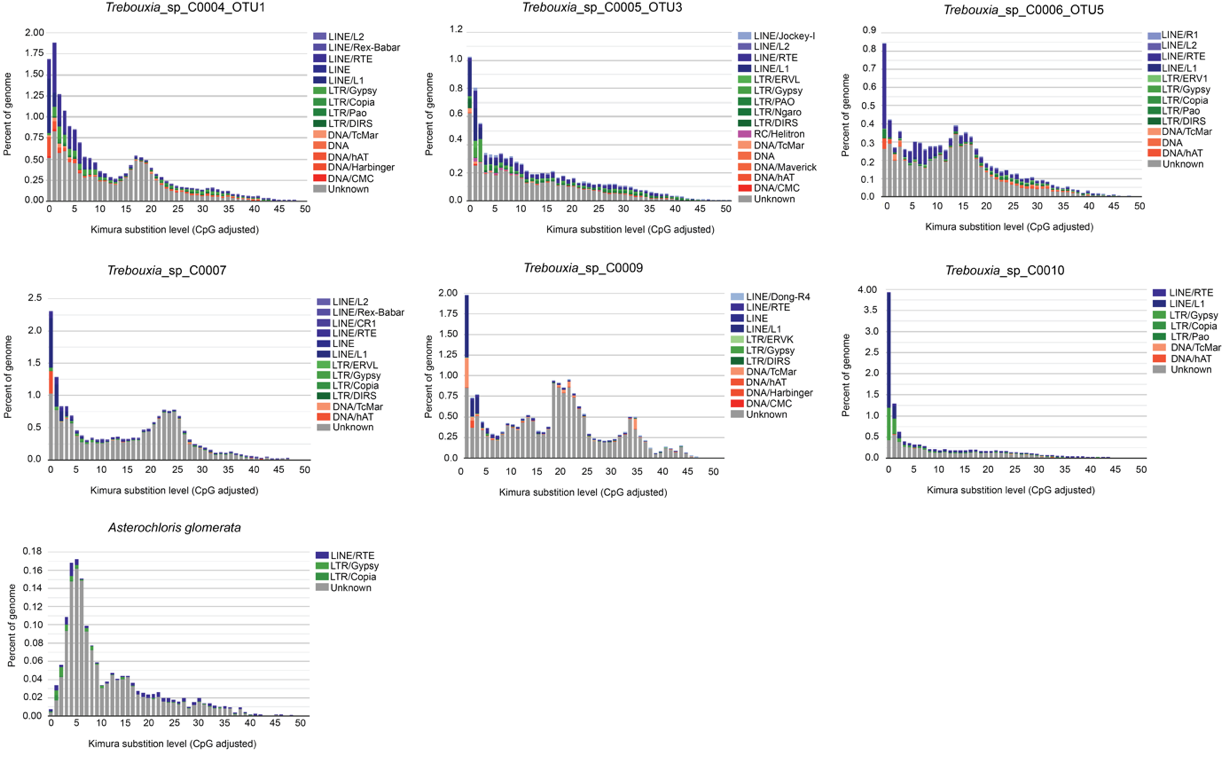


**Supplementary Figure 2** Repeat landscape graphs showing genome coverage (y axis) and copy divergence (x axis, Kimura (K) values from 0-50) of the transposable elements found in the different genomes analysed. Copies on the left (low K values) of the graph represent similar and recent copies while copies on the right (high K values) are highly divergent and represent ancient/degenerated copies. ‘Unknown’ refers to repeats with no matches to known types in the RepeatMasker database.


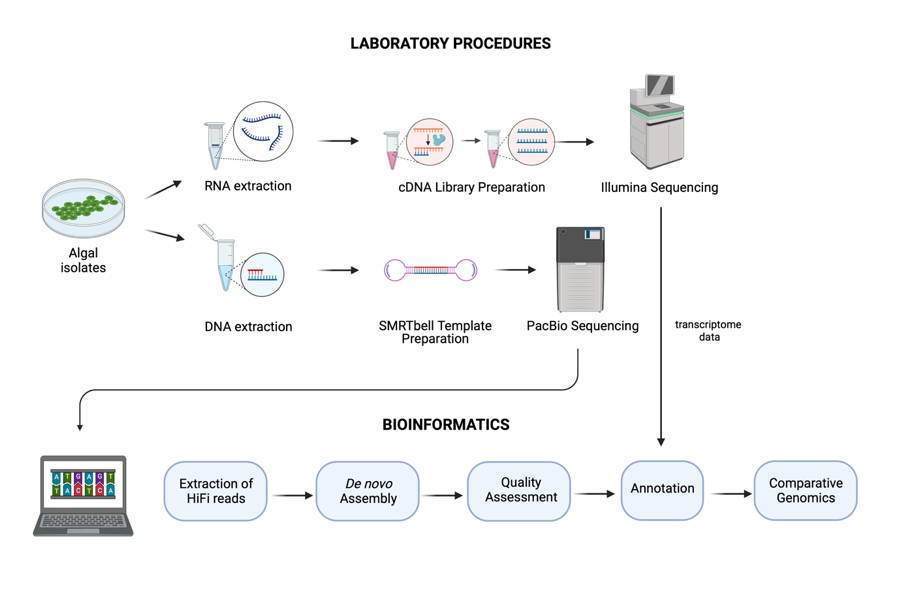
 **Supplementary Figure 3** Summary of laboratory procedures and bioinformatics pipeline. This figure was created using BioRender.com.

**Supplementary Table 2** Evolutionary changes of core gene families, showing the different patterns [gene gains (+) and losses (-), as well as no significant change (0)] across different Trebouxia strains.

| **Gene family** | **S09_C0004** | **S19_C0005** | **S12_C0006** | **A06_C0007** | **A10_C0009** | **A04_C0010** |
| --- | --- | --- | --- | --- | --- | --- |
| OG0000003 | -9 | 12 | 0 | -3 | +41 | -3 |
| OG0000005 | -2 | -1 | 0 | +9 | +3 | 0 |
| OG0000011 | -1 | +6 | 0 | -1 | 0 | -1 |
| OG0000013 | +14 | -1 | -1 | +6 | 1 | -6 |
| OG0000023 | +10 | -1 | 0 | 0 | -3 | +2 |
| OG0000029 | +2 | -2 | +2 | 0 | +1 | 0 |
| OG0000039 | 0 | 0 | +2 | -2 | -1 | +2 |
| OG0000041 | +4 | -2 | 0 | 0 | -5 | 0 |
| OG0000048 | +22 | -2 | 0 | +5 | -2 | 0 |
| OG0000049 | -2 | 0 | +3 | -2 | 0 | 0 |
| OG0000054 | +1 | -3 | +5 | -2 | 0 | 0 |
| OG0000057 | -1 | 0 | 0 | -1 | +8 | -1 |
| OG0000063 | 0 | 0 | +3 | 0 | -1 | 0 |
| OG0000069 | -1 | -1 | +5 | +1 | +2 | -3 |
| OG0000071 | +2 | 0 | 0 | +3 | +1 | -2 |
| OG0000074 | +1 | -5 | +3 | -3 | +3 | 0 |
| OG0000079 | -2 | 0 | +1 | 0 | -1 | +1 |
| OG0000082 | 0 | 0 | 3 | +1 | -1 | +1 |
| OG0000088 | 5 | -5 | +1 | +1 | -1 | 0 |
| OG0000092 | 0 | 0 | 0 | -1 | -1 | +1 |
| OG0000102 | 0 | +1 | -1 | -1 | 0 | 2 |
| OG0000109 | +7 | 0 | -2 | -1 | +2 | -3 |
| OG0000110 | +1 | -1 | +2 | 0 | -1 | 0 |
| OG0000111 | -1 | 14 | -1 | 3 | -1 | 0 |
| OG0000119 | 0 | +3 | 0 | -2 | 0 | +2 |
| OG0000120 | 0 | -3 | +4 | -1 | +6 | -1 |
| OG0000127 | +1 | +1 | 0 | -1 | -1 | 0 |
| OG0000128 | +1 | -3 | 0 | -1 | -1 | +9 |
| OG0000136 | -1 | -1 | +2 | +17 | 0 | -1 |
| OG0000137 | +1 | +1 | -1 | 0 | +8 | -1 |
| OG0000138 | +3 | 0 | -2 | -1 | 0 | +3 |
| OG0000147 | -1 | -2 | +2 | 0 | 0 | +1 |
| OG0000148 | 0 | 0 | 0 | +6 | 0 | 0 |
| OG0000158 | 0 | 0 | -1 | 0 | +8 | -1 |
| OG0000159 | +4 | -4 | 0 | 0 | +3 | -1 |
| OG0000161 | +1 | 0 | -1 | -1 | 0 | +1 |
| OG0000164 | +2 | 0 | -1 | 0 | +1 | 0 |
| OG0000165 | 0 | -1 | +3 | +3 | -2 | 0 |
| OG0000166 | 1 | 0 | -2 | -1 | 2 | 0 |
| OG0000167 | 0 | 0 | -1 | 0 | 4 | 0 |
| OG0000168 | +1 | -1 | 0 | +2 | -3 | +2 |
| OG0000179 | +2 | 0 | -1 | -1 | 0 | -1 |
| OG0000180 | +1 | -1 | 0 | -1 | 0 | -1 |
| OG0000182 | +2 | 0 | -3 | 0 | 0 | 0 |
| OG0000194 | 0 | 0 | +1 | +2 | -1 | +1 |
| OG0000196 | -1 | +1 | -1 | 0 | 0 | +1 |
| OG0000197 | 0 | 0 | 0 | 0 | +3 | -1 |
| OG0000215 | 0 | +5 | -2 | -1 | +1 | 0 |
| OG0000216 | 0 | 0 | +2 | 0 | 0 | 0 |
| OG0000217 | +4 | -1 | 0 | 0 | -1 | +4 |
| OG0000218 | +5 | 0 | -1 | -1 | 0 | 0 |
| OG0000219 | -1 | 0 | 0 | +3 | -3 | +1 |
| OG0000237 | 0 | 0 | -2 | 0 | -2 | 0 |
| OG0000238 | +2 | 0 | 0 | 0 | -1 | 0 |
| OG0000239 | +3 | -1 | 0 | -1 | +4 | 0 |
| OG0000240 | 0 | -1 | +3 | 0 | 0 | 0 |
| OG0000241 | 0 | 0 | +2 | 0 | 0 | 0 |
| OG0000242 | +4 | 0 | 0 | +1 | 0 | 0 |
| OG0000267 | 0 | 0 | +2 | +3 | -1 | 0 |
| OG0000268 | -1 | 1 | 0 | +2 | 0 | 0 |
| OG0000271 | 0 | -1 | +3 | +1 | 0 | 0 |
| OG0000272 | 0 | -2 | +2 | 0 | +1 | -1 |
| OG0000273 | 0 | 0 | 0 | +4 | -1 | 0 |
| OG0000274 | 0 | -2 | 0 | 0 | 0 | -1 |
| OG0000275 | +4 | 0 | -1 | -1 | 0 | 0 |
| OG0000276 | 0 | 0 | +1 | +1 | +1 | -2 |
| OG0000277 | +1 | -2 | +3 | -1 | 0 | -1 |
| OG0000278 | 0 | +1 | 0 | -1 | -1 | 3 |
| OG0000279 | +1 | -1 | 0 | 0 | +1 | 0 |
| OG0000281 | 0 | +3 | -2 | 0 | -1 | 1 |
| OG0000282 | 0 | -2 | 0 | 0 | 0 | -1 |
| OG0000283 | +1 | -2 | +1 | 0 | +1 | 0 |
| OG0000285 | +1 | -1 | +1 | -1 | -1 | +1 |
| OG0000306 | 0 | 2 | 0 | -1 | 0 | +1 |
| OG0000307 | 0 | 0 | -1 | -1 | 0 | +1 |
| OG0000308 | 0 | -2 | 0 | -1 | 0 | +3 |
| OG0000309 | 0 | +1 | 0 | 0 | 0 | +1 |
| OG0000310 | 0 | +3 | 0 | 0 | 0 | +1 |
| OG0000316 | 0 | +3 | 0 | 0 | -1 | +1 |
| OG0000317 | 0 | +4 | 0 | 0 | -1 | 0 |
| OG0000321 | 0 | -2 | +1 | 0 | 0 | -2 |
| OG0000322 | +2 | -1 | 0 | 0 | -2 | 0 |
| OG0000323 | 0 | 0 | +6 | 0 | 0 | +1 |
| OG0000324 | 0 | -2 | +1 | -1 | 0 | +2 |
| OG0000326 | 0 | +1 | 0 | -1 | +1 | 0 |
| OG0000327 | +2 | 0 | -1 | -1 | -1 | +1 |
| OG0000328 | 0 | +2 | 0 | +2 | -1 | 0 |
| OG0000329 | +1 | +2 | -3 | 0 | +2 | -2 |
| OG0000330 | 0 | 0 | 0 | 0 | 0 | -1 |
| OG0000336 | -1 | -1 | +1 | 0 | -1 | +2 |
| OG0000338 | 0 | -2 | +1 | 0 | +1 | 0 |
| OG0000339 | 0 | +2 | 0 | 0 | -1 | 0 |
| OG0000340 | -1 | 0 | +1 | +2 | -2 | 0 |
| OG0000341 | -1 | +4 | 0 | -2 | 0 | 0 |
| OG0000342 | 0 | -1 | +2 | -1 | 0 | 0 |
| OG0000343 | 0 | -1 | +2 | 0 | 0 | -1 |
| OG0000382 | +2 | 0 | 0 | 0 | 0 | 0 |
| OG0000384 | 0 | -1 | 0 | -1 | 0 | +2 |
| OG0000385 | +1 | -2 | +1 | +1 | 0 | 0 |
| OG0000388 | 0 | +2 | -1 | 0 | -1 | +2 |
| OG0000389 | +2 | 0 | 0 | 0 | -2 | 0 |
| OG0000393 | 0 | -1 | 0 | +2 | -2 | 0 |
| OG0000394 | 0 | 0 | -1 | +3 | 0 | 0 |
| OG0000395 | 0 | 4 | -1 | 0 | -1 | 0 |
| OG0000396 | 0 | +2 | 0 | -1 | +1 | 0 |
| OG0000399 | +2 | 0 | 0 | 0 | -1 | 0 |
| OG0000402 | 0 | -1 | 0 | -1 | 0 | 0 |
| OG0000406 | 0 | -1 | +3 | 0 | 0 | 0 |
| OG0000407 | 0 | 0 | -2 | -1 | +3 | -1 |
| OG0000411 | +1 | 0 | -1 | -1 | +1 | 0 |
| OG0000412 | 0 | 0 | +4 | 0 | 0 | +2 |
| OG0000413 | +5 | -1 | 0 | 0 | 0 | +1 |
| OG0000414 | 0 | +2 | -1 | 0 | -1 | +2 |
| OG0000417 | +3 | 0 | 0 | +1 | 0 | 0 |
| OG0000420 | 0 | +2 | 0 | 0 | 0 | +1 |
| OG0000421 | 0 | +1 | -2 | -1 | -1 | +1 |
| OG0000422 | +1 | +1 | -3 | -1 | 0 | +1 |
| OG0000488 | 0 | +1 | 0 | +5 | 0 | 0 |
| OG0000491 | +1 | -2 | +1 | 0 | 0 | +1 |
| OG0000494 | +1 | -1 | 0 | 0 | 2 | -1 |
| OG0000495 | 0 | 0 | -1 | -1 | -1 | +1 |
| OG0000496 | 0 | 0 | +2 | 0 | +1 | 0 |
| OG0000498 | 0 | -2 | +2 | 0 | +1 | 0 |
| OG0000501 | 0 | -1 | +2 | -1 | 0 | +1 |
| OG0000504 | -1 | +1 | 0 | 0 | -1 | 0 |
| OG0000506 | 0 | 0 | +2 | +1 | 0 | 0 |
| OG0000508 | +3 | 0 | -2 | 0 | 0 | 0 |
| OG0000509 | 0 | +2 | -1 | -1 | 0 | +1 |
| OG0000512 | +1 | 0 | 0 | 0 | -2 | +1 |
| OG0000513 | +2 | 0 | -2 | 0 | 0 | +1 |
| OG0000514 | 0 | +3 | 0 | 0 | 0 | +2 |
| OG0000515 | +1 | 0 | -2 | +2 | -1 | 0 |
| OG0000516 | +2 | 0 | -1 | 0 | +1 | -1 |
| OG0000519 | 0 | -1 | +1 | 0 | +1 | 0 |
| OG0000522 | 0 | -1 | +1 | 0 | +1 | 0 |
| OG0000526 | 0 | -1 | +3 | 0 | 0 | +2 |
| OG0000529 | +1 | -1 | 0 | -1 | -1 | +1 |
| OG0000531 | 0 | 0 | +1 | +1 | -1 | 0 |
| OG0000534 | 0 | -1 | +4 | +2 | 0 | 0 |
| OG0000535 | +3 | 0 | 0 | -1 | 0 | 0 |
| OG0000536 | 0 | +2 | 0 | +1 | +1 | 0 |
| OG0000537 | 0 | 0 | +4 | 0 | 0 | 0 |
| OG0000538 | +2 | 0 | 0 | 0 | 0 | +2 |
| OG0000539 | 0 | +2 | 0 | 0 | 0 | +1 |
| OG0000541 | 0 | +3 | 0 | 0 | 0 | +2 |
| OG0000543 | +2 | -3 | 0 | +1 | 0 | 0 |
| OG0000544 | +1 | -2 | 0 | -1 | 0 | 0 |
| OG0000549 | 0 | -2 | 0 | -1 | +1 | 0 |
| OG0000550 | +3 | 0 | -1 | 0 | 0 | +2 |
| OG0000551 | +1 | 0 | -1 | -1 | 0 | 0 |
| OG0000552 | -1 | -1 | +2 | +1 | -1 | +1 |
| OG0000554 | +1 | 0 | 0 | 0 | 0 | +4 |
| OG0000556 | 0 | -1 | +1 | 0 | 0 | +1 |
| OG0000558 | -1 | +1 | 0 | -1 | +2 | 0 |
| OG0000560 | +1 | +1 | -2 | 0 | 0 | +1 |
| OG0000561 | 0 | 0 | +5 | 0 | 0 | +2 |
| OG0000564 | +6 | 0 | 0 | 0 | 0 | 0 |
| OG0000565 | -1 | 0 | 0 | 0 | +1 | -1 |
| OG0000567 | -1 | 0 | 0 | +3 | 0 | -1 |
| OG0000629 | -1 | 0 | +2 | 0 | 0 | -1 |
| OG0000630 | 0 | -1 | +2 | +1 | 0 | 0 |
| OG0000633 | +1 | 0 | -1 | 0 | -1 | +1 |
| OG0000635 | 0 | +3 | 0 | 0 | 0 | 0 |
| OG0000636 | 0 | +2 | -1 | 0 | -1 | 0 |
| OG0000637 | 0 | +4 | -1 | +4 | 0 | 0 |
| OG0000641 | +1 | -1 | 0 | 0 | +1 | -1 |
| OG0000642 | -1 | +2 | 0 | 0 | -1 | 0 |
| OG0000645 | +1 | 0 | 0 | +1 | +1 | -1 |
| OG0000646 | 0 | +2 | 0 | 0 | 0 | 0 |
| OG0000648 | 0 | +2 | 0 | 0 | +1 | 0 |
| OG0000650 | +1 | 0 | 0 | 0 | -1 | +2 |
| OG0000651 | 0 | 0 | -1 | -1 | -1 | +3 |
| OG0000652 | +2 | 0 | -1 | 0 | -1 | 0 |
| OG0000653 | 0 | +3 | 0 | +3 | 0 | 0 |
| OG0000654 | -1 | -1 | +2 | 0 | -1 | +1 |
| OG0000655 | 0 | +4 | 0 | 0 | 0 | 0 |
| OG0000656 | 0 | +6 | 0 | 0 | 0 | 0 |
| OG0000657 | 0 | +1 | -1 | 0 | -1 | +1 |
| OG0000658 | 0 | 0 | 0 | +4 | -1 | 0 |
| OG0000663 | 0 | +2 | 0 | 0 | 0 | 0 |
| OG0000665 | +1 | +1 | -1 | 0 | +2 | 0 |
| OG0000670 | +2 | -1 | 0 | -1 | 0 | 0 |
| OG0000672 | 0 | +3 | 0 | +1 | 0 | 0 |
| OG0000673 | -1 | 0 | +2 | 0 | -1 | 0 |
| OG0000674 | +2 | 0 | 0 | +2 | 0 | 0 |
| OG0000675 | -1 | +3 | 0 | 0 | 0 | 0 |
| OG0000676 | 0 | 0 | 0 | +1 | -2 | 0 |
| OG0000682 | +5 | 0 | 0 | +1 | 0 | 0 |
| OG0000683 | 0 | -1 | +2 | 0 | 0 | +2 |
| OG0000688 | 0 | -2 | +1 | 0 | + | 0 |
| OG0000689 | +1 | 0 | 0 | 0 | -1 | +2 |
| OG0000690 | 0 | +1 | -2 | 0 | 0 | +1 |
| OG0000691 | +3 | 0 | -1 | 0 | 0 | +1 |
| OG0000693 | +1 | 0 | -2 | 0 | 0 | +1 |
| OG0000696 | +3 | 0 | 0 | 0 | -1 | 0 |
| OG0000701 | 0 | +3 | -1 | 0 | 0 | 0 |
| OG0000702 | 0 | -1 | +1 | -1 | -1 | +2 |
| OG0000708 | -2 | 0 | +1 | -1 | 0 | 0 |
| OG0000709 | +2 | -1 | 0 | 0 | +2 | 0 |
| OG0000710 | 0 | 0 | +5 | 0 | +1 | 0 |
| OG0000711 | 0 | 0 | +3 | 0 | 0 | +3 |
| OG0000712 | 0 | -1 | +3 | 0 | 0 | +1 |
| OG0000713 | +2 | -1 | 0 | -1 | 0 | 0 |
| OG0000717 | +2 | 0 | 0 | -1 | 0 | -1 |
| OG0000718 | +1 | 0 | 0 | 0 | -1 | +2 |
| OG0000721 | 0 | 0 | -1 | -1 | +2 | 0 |
| OG0000722 | 0 | +3 | 0 | 0 | +1 | 0 |
| OG0000725 | 0 | +6 | 0 | 0 | 0 | 0 |
| OG0000726 | 0 | 0 | 0 | +4 | -1 | 0 |
| OG0000728 | 0 | +1 | 0 | 0 | 0 | +3 |
| OG0000729 | -1 | 0 | +1 | -1 | +1 | 0 |
| OG0000735 | 0 | 0 | +2 | 0 | 0 | 0 |
| OG0000736 | 0 | 0 | +1 | +2 | -1 | +1 |
| OG0000741 | +1 | +1 | -1 | 0 | +2 | 0 |
| OG0000743 | 0 | 0 | -1 | -1 | 0 | +2 |
| OG0000746 | +2 | 0 | 0 | 0 | 0 | +4 |
| OG0000752 | 0 | +2 | -1 | 0 | 0 | -1 |
| OG0000753 | -1 | 0 | 0 | +1 | 0 | 0 |
| OG0000754 | -1 | -1 | +2 | +1 | -1 | 0 |
| OG0000756 | 0 | 0 | +2 | 0 | 0 | +2 |
| OG0000758 | 0 | -1 | +1 | 0 | -1 | +1 |
| OG0000761 | +1 | -1 | 0 | 0 | -1 | +1 |
| OG0000762 | 0 | 1 | -1 | -1 | -1 | 2 |
| OG0000764 | +2 | 0 | -1 | -1 | 0 | 0 |
| OG0000765 | -1 | 0 | 0 | +1 | 0 | 0 |
| OG0000767 | +1 | 0 | -2 | 0 | 0 | 0 |
| OG0000839 | +2 | -1 | 0 | 0 | +1 | 0 |
| OG0000842 | +1 | +1 | -1 | 0 | +1 | 0 |
| OG0000844 | 0 | +1 | 0 | +4 | 0 | 0 |
| OG0000850 | +1 | -2 | 0 | -1 | 0 | 0 |
| OG0000851 | 0 | +2 | -1 | 0 | +1 | 0 |
| OG0000852 | 0 | +2 | 0 | +1 | 0 | -1 |
| OG0000855 | 0 | -1 | +1 | 0 | +2 | 0 |
| OG0000857 | 0 | 0 | +3 | 0 | 0 | 0 |
| OG0000858 | 0 | 0 | 0 | 0 | 0 | +3 |
| OG0000859 | 0 | +2 | 0 | 0 | 0 | +1 |
| OG0000860 | 0 | 0 | +1 | 0 | +2 | -1 |
| OG0000861 | 0 | -1 | +1 | 0 | 0 | +2 |
| OG0000863 | 0 | 0 | +3 | 0 | 0 | +2 |
| OG0000864 | 0 | 0 | +1 | 0 | 0 | +2 |
| OG0000868 | +1 | 0 | 0 | 0 | 0 | +4 |
| OG0000869 | 0 | 0 | +1 | +2 | 0 | -1 |
| OG0000872 | +2 | 0 | -1 | 0 | 0 | +1 |
| OG0000874 | +3 | 0 | 0 | +2 | 0 | 0 |
| OG0000875 | 0 | 0 | +2 | +1 | 0 | -1 |
| OG0000876 | +1 | 0 | 0 | 0 | 0 | +4 |
| OG0000878 | +2 | -1 | 0 | 0 | +1 | 0 |
| OG0000879 | 0 | +1 | -1 | 0 | 0 | +2 |
| OG0000880 | +1 | -2 | 0 | 0 | 0 | 0 |
| OG0000883 | +1 | 0 | 0 | +1 | -1 | 0 |
| OG0000884 | 0 | 0 | +1 | 0 | +2 | -1 |
| OG0000886 | +1 | +1 | -1 | 0 | 0 | +1 |
| OG0000887 | +2 | -1 | 0 | 0 | 0 | +1 |
| OG0000888 | 0 | +1 | 0 | 0 | -1 | +2 |
| OG0000889 | 0 | 0 | 0 | 0 | +5 | 0 |
| OG0000895 | 0 | 0 | +1 | 0 | -1 | +2 |
| OG0000900 | +1 | 0 | 0 | 0 | 0 | +2 |
| OG0000901 | 0 | 0 | +3 | 0 | 0 | 0 |
| OG0000907 | +1 | -1 | 0 | 0 | +2 | 0 |
| OG0000908 | 0 | 0 | +2 | 0 | 0 | +1 |
| OG0000909 | 0 | +1 | -1 | 0 | 0 | +2 |
| OG0000912 | 0 | -1 | 0 | -1 | -1 | +2 |
| OG0000914 | 0 | +3 | -1 | 0 | 0 | 0 |
| OG0000915 | 0 | +3 | 0 | 0 | 0 | 0 |
| OG0000916 | 0 | 0 | +2 | 0 | +1 | 0 |
| OG0000918 | +1 | 0 | 0 | +1 | -1 | 0 |
| OG0000919 | 0 | 0 | +4 | 0 | 0 | +1 |
| OG0000926 | +2 | -1 | 0 | 0 | 0 | 0 |
| OG0000928 | 0 | 0 | +3 | 0 | +1 | 0 |
| OG0000930 | +4 | 0 | 0 | +1 | 0 | 0 |
| OG0000936 | 0 | +1 | -1 | 0 | 0 | +2 |
| OG0000938 | +1 | -1 | +1 | 0 | 0 | +1 |
| OG0000940 | +1 | -1 | 0 | 0 | 0 | +2 |
| OG0000943 | 0 | 0 | 0 | +2 | -1 | 0 |
| OG0000951 | 0 | 0 | +3 | 0 | 0 | 0 |
| OG0000956 | +3 | 0 | 0 | 0 | 0 | +1 |
| OG0000957 | 0 | 0 | +3 | +2 | 0 | 0 |
| OG0000958 | +1 | -1 | +1 | 0 | +1 | 0 |
| OG0000960 | +1 | -1 | +1 | 0 | 0 | +1 |
| OG0000961 | 0 | 0 | 0 | +2 | 0 | -1 |
| OG0000962 | 0 | +1 | 0 | +1 | -1 | +1 |
| OG0000966 | +1 | +1 | -1 | 0 | 0 | +1 |
| OG0000967 | 0 | +2 | 0 | 0 | 0 | +1 |
| OG0000970 | +1 | +1 | -1 | 0 | +1 | 0 |
| OG0000972 | 2 | -1 | 0 | 0 | 0 | 0 |
| OG0000973 | 0 | +4 | 0 | 0 | 0 | +1 |
| OG0000974 | 0 | 0 | +2 | +1 | 0 | 0 |
| OG0000976 | +4 | 0 | 0 | 0 | 0 | +1 |
| OG0000979 | +4 | 0 | 0 | 0 | 0 | +1 |
| OG0000980 | +1 | 0 | -1 | 0 | 0 | +2 |
| OG0000981 | +3 | -1 | 0 | 0 | 0 | 0 |
| OG0000983 | -1 | -1 | 2 | 0 | -1 | 0 |
| OG0000985 | +2 | 0 | -1 | 0 | 0 | 0 |
| OG0000986 | 0 | 0 | +2 | +1 | 0 | 0 |
| OG0000988 | 0 | +1 | 0 | 0 | 0 | +4 |
| OG0000989 | 0 | 0 | 0 | +1 | -1 | +2 |
| OG0000990 | 0 | +1 | 0 | +1 | -1 | +1 |
| OG0000991 | +1 | -1 | +1 | 0 | 0 | +1 |
| OG0000998 | 0 | +1 | 0 | 0 | +2 | 0 |
| OG0000999 | +3 | 0 | 0 | 0 | 0 | +2 |
| OG0001001 | 0 | +3 | 0 | 0 | 0 | 0 |
| OG0001006 | 0 | +2 | 0 | 0 | -1 | +1 |
| OG0001007 | 0 | +2 | 0 | 0 | 0 | +1 |
| OG0001009 | 0 | +2 | 0 | 0 | +1 | 0 |
| OG0001014 | +1 | 0 | 0 | +1 | 0 | -1 |
| OG0001015 | 0 | -1 | +1 | +1 | 0 | 0 |
| OG0001016 | 0 | +1 | -2 | 0 | 0 | 0 |
| OG0001020 | 0 | 0 | 0 | 0 | -1 | +3 |
| OG0001021 | 0 | 0 | +2 | +1 | 0 | 0 |
| OG0001023 | +1 | 0 | -1 | 0 | 0 | +2 |
| OG0001025 | 0 | +2 | -1 | 0 | +1 | 0 |
| OG0001026 | 0 | 0 | 0 | +1 | -1 | +2 |
| OG0001027 | 0 | -2 | +2 | 0 | -1 | 0 |
| OG0001028 | 0 | 0 | 0 | 0 | +3 | 0 |
| OG0001031 | +2 | 0 | 0 | -1 | 0 | 0 |
| OG0001169 | +1 | 0 | 0 | 0 | 0 | +3 |
| OG0001173 | 0 | 0 | +3 | +1 | 0 | 0 |
| OG0001181 | -1 | +1 | 0 | 0 | 0 | +1 |
| OG0001184 | +3 | 0 | 0 | 0 | 0 | +1 |
| OG0001191 | +1 | 0 | 0 | 0 | 0 | +3 |
| OG0001192 | 0 | +2 | 0 | 0 | 0 | +2 |
| OG0001196 | +2 | 0 | 0 | +2 | 0 | 0 |
| OG0001206 | +1 | 0 | 0 | 0 | 0 | +3 |
| OG0001213 | +1 | 0 | 0 | +3 | 0 | 0 |
| OG0001225 | +2 | 0 | 0 | +2 | 0 | 0 |
| OG0001244 | +4 | 0 | -1 | 0 | 0 | 0 |
| OG0001247 | 0 | 0 | +4 | 0 | 0 | 0 |
| OG0001248 | 0 | +2 | 0 | 0 | +2 | 0 |
| OG0001253 | 0 | 0 | +4 | 0 | 0 | 0 |
| OG0001256 | +3 | 0 | 0 | 0 | 0 | +1 |
| OG0001258 | 0 | +1 | -1 | +1 | -1 | +1 |
| OG0001260 | 0 | 0 | +3 | +1 | 0 | 0 |
| OG0001264 | 0 | 0 | 0 | 0 | +4 | 0 |
| OG0001266 | 0 | 0 | +4 | 0 | 0 | 0 |
| OG0001275 | 0 | 0 | 0 | -1 | +1 | -1 |
| OG0001279 | 0 | +3 | 0 | +2 | 0 | -1 |
| OG0001299 | +1 | 0 | 0 | 0 | +3 | 0 |
| OG0001301 | +3 | 0 | 0 | +1 | 0 | 0 |
| OG0001308 | 0 | +1 | 0 | 0 | 0 | +3 |
| OG0001312 | 0 | 0 | 4 | 0 | 0 | 0 |
| OG0001333 | +1 | 0 | 0 | 0 | 0 | +3 |
| OG0001335 | 0 | +2 | 0 | 0 | +2 | 0 |
| OG0001337 | 0 | +1 | 0 | 0 | 0 | +3 |
| OG0001340 | 0 | +3 | 0 | +1 | 0 | 0 |
| OG0001345 | 0 | +3 | 0 | 0 | 0 | +1 |
| OG0001351 | 0 | +4 | 0 | 0 | 0 | 0 |
| OG0001353 | 0 | +2 | 0 | +2 | 0 | 0 |
| OG0001366 | 0 | 0 | +3 | 0 | 0 | +1 |
| OG0001367 | -1 | 0 | 0 | +4 | 0 | 0 |
| OG0001385 | 0 | +1 | 0 | 0 | 0 | +3 |
| OG0001386 | 0 | +3 | 0 | 0 | 0 | +1 |
| OG0001400 | 0 | 0 | +4 | 0 | 0 | 0 |
| OG0001401 | +2 | 0 | 0 | 0 | 0 | +2 |
| OG0001402 | +3 | 0 | 0 | 0 | +1 | 0 |
| OG0001403 | 0 | 0 | +3 | +1 | 0 | 0 |
| OG0001405 | +1 | -1 | 0 | 0 | -2 | 0 |
| OG0001418 | 0 | 0 | +4 | 0 | 0 | 0 |
| OG0001424 | 0 | 0 | +4 | 0 | 0 | 0 |
| OG0001425 | +3 | 0 | 0 | 0 | +1 | 0 |
| OG0001426 | 0 | 0 | +4 | 0 | 0 | 0 |
| OG0001429 | +2 | 0 | 0 | 0 | 0 | -1 |
| OG0001436 | 0 | +2 | 0 | 0 | +2 | 0 |
| OG0001439 | 0 | 0 | 0 | +4 | 0 | 0 |
| OG0001441 | 0 | +1 | 0 | 0 | 0 | +3 |
| OG0001449 | 0 | +2 | 0 | +2 | 0 | 0 |
| OG0001450 | 0 | 0 | +3 | 0 | +1 | 0 |
| OG0001460 | 0 | +2 | 0 | 0 | +2 | 0 |
| OG0001461 | +4 | 0 | 0 | 0 | 0 | 0 |
| OG0001467 | 0 | 0 | 0 | 0 | 0 | +4 |
| OG0001471 | +4 | 0 | 0 | 0 | 0 | 0 |
| OG0001480 | 0 | -2 | +3 | 0 | 0 | 0 |
| OG0001481 | -1 | 0 | +2 | 0 | -1 | +1 |
| OG0001484 | +2 | 0 | 0 | +2 | 0 | 0 |
| OG0001485 | -1 | -1 | 0 | +1 | -2 | +1 |
| OG0001501 | +3 | 0 | 0 | 0 | 0 | +1 |
| OG0001502 | 0 | +2 | 0 | +2 | 0 | 0 |
| OG0001503 | 0 | 0 | +3 | 0 | +1 | 0 |
| OG0001505 | +3 | 0 | 0 | 0 | +1 | 0 |
| OG0001513 | 0 | 0 | +1 | 0 | +3 | 0 |
| OG0001515 | +3 | 0 | 0 | 0 | +1 | 0 |
| OG0001516 | 0 | -1 | +4 | 0 | 0 | +1 |
| OG0001518 | +1 | 0 | 0 | 0 | 0 | +3 |
| OG0001519 | 0 | -2 | +1 | 0 | 0 | -1 |
| OG0001714 | 0 | +3 | 0 | 0 | 0 | 0 |
| OG0001720 | 0 | +1 | 0 | 0 | -2 | +1 |
| OG0001721 | 0 | 0 | +3 | 0 | 0 | 0 |
| OG0001743 | +1 | 0 | -1 | 0 | 0 | 0 |
| OG0001769 | 0 | 0 | +3 | 0 | 0 | 0 |
| OG0001776 | +1 | 0 | -1 | 0 | +3 | 0 |
| OG0001817 | 0 | 0 | -1 | +3 | 0 | 0 |
| OG0001820 | 0 | 0 | +3 | 0 | 0 | 0 |
| OG0001851 | 0 | -1 | +5 | -1 | 0 | -1 |
| OG0001901 | 0 | 0 | +3 | 0 | 0 | 0 |
| OG0001917 | 0 | 0 | -1 | 0 | -1 | +2 |
| OG0001954 | 0 | 0 | +3 | 0 | 0 | 0 |
| OG0001960 | 0 | +2 | -1 | 0 | 0 | 0 |
| OG0001991 | 0 | +1 | 0 | -1 | 0 | +3 |
| OG0001993 | 0 | 0 | +2 | 0 | -2 | 0 |
| OG0002017 | 0 | 0 | +3 | 0 | 0 | 0 |
| OG0002028 | 0 | +1 | 0 | 0 | -2 | +1 |
| OG0002036 | +2 | -1 | 0 | -1 | +4 | -1 |
| OG0002057 | 0 | 0 | +3 | 0 | 0 | 0 |
| OG0002060 | +2 | -1 | 0 | -1 | 0 | 0 |
| OG0002136 | 0 | +3 | 0 | 0 | 0 | 0 |
| OG0002150 | 0 | 0 | +3 | 0 | 0 | 0 |
| OG0002166 | 0 | 0 | -1 | +1 | -1 | +1 |
| OG0002167 | 0 | 0 | +3 | 0 | 0 | 0 |
| OG0002199 | 0 | -1 | +3 | 0 | +2 | -1 |
| OG0002207 | 0 | +2 | -1 | 0 | 0 | +2 |
| OG0002231 | 0 | 0 | +3 | 0 | 0 | 0 |
| OG0002237 | 0 | +3 | 0 | 0 | 0 | 0 |
| OG0002239 | +1 | 0 | -2 | 0 | 0 | 0 |
| OG0002617 | 0 | 0 | +2 | +2 | 0 | 0 |
| OG0002681 | 0 | -1 | 0 | 0 | -1 | +1 |
| OG0002689 | -1 | 0 | 0 | 0 | 0 | +3 |
| OG0002731 | +1 | -1 | 0 | 0 | 0 | +2 |
| OG0002756 | +3 | 0 | 0 | 0 | -1 | 0 |
| OG0002814 | +2 | 0 | -1 | 0 | +1 | 0 |
| OG0002867 | 0 | +1 | -1 | 0 | -1 | 0 |
| OG0002987 | 0 | -2 | 0 | 0 | 0 | +1 |
| OG0002997 | 0 | 0 | 0 | 0 | +1 | -2 |
| OG0003029 | 0 | 0 | -2 | 0 | +1 | 0 |
| OG0003068 | +3 | 0 | -1 | 0 | 0 | 0 |
| OG0003115 | +3 | 0 | 0 | 0 | 0 | -1 |
| OG0003194 | +2 | -1 | 0 | 0 | +1 | 0 |
| OG0003220 | 0 | 0 | +2 | -1 | 0 | +1 |
| OG0003225 | +1 | -1 | 0 | -1 | +3 | 0 |
| OG0003282 | 0 | +1 | -1 | +2 | 0 | 0 |
| OG0003840 | 0 | 0 | +2 | 0 | 0 | -1 |
| OG0003903 | +5 | 0 | 0 | 0 | 0 | 0 |
| OG0004401 | 0 | -1 | +1 | 0 | +1 | 0 |
| OG0004479 | 0 | 0 | 0 | 0 | +4 | -1 |
| OG0004530 | 0 | +2 | 0 | 0 | -1 | 0 |
| OG0004602 | 0 | +2 | -1 | 0 | 0 | 0 |
| OG0004657 | 0 | +2 | -1 | 0 | +1 | -1 |
| OG0004744 | +3 | -1 | 0 | 0 | -1 | 0 |
| OG0004841 | +3 | 0 | -1 | +1 | 0 | 0 |
| OG0004949 | -1 | 0 | +1 | 0 | 0 | -1 |
| OG0005001 | 0 | +2 | 0 | 0 | 0 | -1 |
| OG0005954 | 0 | 0 | -1 | -1 | -1 | 0 |
| OG0005996 | 0 | -1 | 0 | -1 | +2 | 0 |
| OG0006003 | 0 | +1 | -1 | +1 | 0 | -1 |
| OG0006112 | -1 | 0 | 0 | -1 | 0 | +2 |
| OG0006432 | -1 | -1 | 0 | 0 | +2 | 0 |
| OG0006590 | -1 | 1 | -1 | 0 | 0 | +1 |
| OG0006821 | +1 | 0 | 0 | -1 | +1 | -1 |
| OG0006939 | 0 | 0 | -1 | -1 | 0 | +2 |
| OG0006940 | -1 | 0 | -1 | 0 | -1 | 0 |
| OG0008262 | -1 | +1 | -1 | 0 | 0 | 0 |
| OG0008329 | +3 | 0 | 0 | -1 | -1 | 0 |
| OG0008335 | -1 | 0 | -1 | -1 | +2 | 0 |
| OG0008363 | -1 | 0 | 0 | -1 | 0 | -1 |
| OG0008371 | 0 | 0 | 0 | +4 | 0 | 0 |
| OG0008403 | -1 | -1 | +1 | 0 | 0 | 0 |
| OG0008436 | -1 | -1 | +1 | 0 | 0 | 0 |
| OG0009786 | 0 | 0 | 0 | 0 | -1 | 2 |
| OG0009788 | 0 | 0 | 0 | 0 | +1 | -1 |
| OG0009820 | 0 | -1 | +1 | -1 | 0 | -1 |
| OG0009822 | -1 | 0 | 2 | 0 | 0 | 0 |
| OG0009826 | 0 | 0 | -1 | -1 | +1 | -1 |
| OG0009831 | 0 | -1 | 0 | -1 | -1 | +1 |
| OG0009841 | 0 | -1 | 0 | -1 | +1 | -1 |
| OG0009842 | -1 | 0 | -1 | 0 | -1 | +1 |
| OG0009850 | -1 | +2 | -1 | 0 | 0 | 0 |
| OG0009861 | 0 | -1 | +1 | 0 | 0 | 0 |
| OG0009863 | -1 | 0 | +2 | 0 | 0 | 0 |
| OG0010502 | 0 | -1 | 0 | -1 | -1 | 0 |
| OG0010520 | +1 | 0 | 0 | -1 | -1 | 0 |
| OG0010523 | 0 | -1 | 0 | -1 | 0 | -1 |
| OG0010525 | -1 | -1 | 0 | 0 | -1 | 0 |
| OG0010530 | -1 | 0 | -1 | -1 | 0 | 0 |
| OG0010532 | 0 | 0 | -1 | -1 | -1 | 0 |
| OG0010533 | -1 | -1 | 0 | -1 | 0 | 0 |
| OG0010540 | -1 | -1 | 0 | -1 | 0 | 0 |
| OG0010542 | -1 | 0 | -1 | 0 | 0 | -1 |
| OG0010551 | -1 | 0 | -1 | 0 | 0 | -1 |
| OG0010554 | -1 | -1 | 1 | -1 | -1 | 0 |
| OG0011256 | 0 | 0 | 0 | -1 | -1 | 0 |
| OG0011259 | -1 | 0 | -1 | -1 | 0 | -1 |
| OG0011263 | 0 | 0 | 0 | -1 | 0 | -1 |
| OG0011267 | -1 | -1 | 0 | -1 | 0 | -1 |
| OG0011269 | -1 | -1 | 1 | 0 | 0 | 0 |
| OG0011270 | -1 | 0 | -1 | -1 | -1 | 0 |
| OG0011281 | 0 | 0 | 0 | -1 | -1 | 0 |
| OG0011282 | -1 | -1 | 0 | -1 | 0 | -1 |
| OG0011288 | -1 | -1 | 0 | -1 | -1 | 0 |
| OG0011297 | 0 | 0 | 0 | -1 | +1 | -1 |
| OG0011300 | -1 | 0 | -1 | -1 | -1 | 0 |
| OG0011304 | -1 | 0 | -1 | -1 | -1 | 0 |
| OG0011308 | 0 | 0 | 0 | -1 | 0 | -1 |
